# Soft, Multifunctional MXene-Coated Fiber Microelectrodes for Biointerfacing

**DOI:** 10.1021/acsnano.4c05797

**Published:** 2024-08-14

**Authors:** Lingyi Bi, Raghav Garg, Natalia Noriega, Ruocun John Wang, Hyunho Kim, Kseniia Vorotilo, Justin C. Burrell, Christopher E. Shuck, Flavia Vitale, Bhavik Anil Patel, Yury Gogotsi

**Affiliations:** † Department of Materials Science and Engineering and A. J. Drexel Nanomaterials Institute, Drexel University, Philadelphia, Pennsylvania 19104, United States; ‡ Department of Neurology, University of Pennsylvania, Philadelphia, Pennsylvania 19104, United States; § School of Applied Sciences, University of Brighton, Brighton BN2 4AT, U.K.; ∥ Department of Bioengineering, University of Pennsylvania, Philadelphia, Pennsylvania 19104, United States; ⊥ Department of Physical Medicine and Rehabilitation, University of Pennsylvania, Philadelphia, Pennsylvania 19104, United States; # Department of Oral and Maxillofacial Surgery & Pharmacology, University of Pennsylvania School of Dental Medicine, Philadelphia, Pennsylvania 19104, United States

**Keywords:** dip coating, fiber electrode, chemical sensing, electrical sensing, neural stimulation, MXene

## Abstract

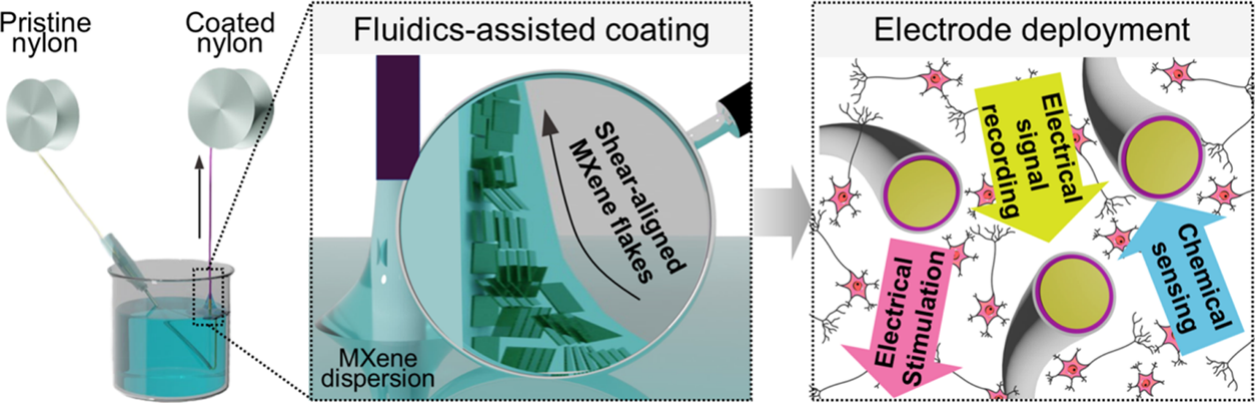

Flexible fiber-based microelectrodes allow safe and chronic investigation and modulation of electrically active cells and tissues. Compared to planar electrodes, they enhance targeting precision while minimizing side effects from the device-tissue mechanical mismatch. However, the current manufacturing methods face scalability, reproducibility, and handling challenges, hindering large-scale deployment. Furthermore, only a few designs can record electrical and biochemical signals necessary for understanding and interacting with complex biological systems. In this study, we present a method that utilizes the electrical conductivity and easy processability of MXenes, a diverse family of two-dimensional nanomaterials, to apply a thin layer of MXene coating continuously to commercial nylon filaments (30–300 μm in diameter) at a rapid speed (up to 15 mm/s), achieving a linear resistance below 10 Ω/cm. The MXene-coated filaments are then batch-processed into free-standing fiber microelectrodes with excellent flexibility, durability, and consistent performance even when knotted. We demonstrate the electrochemical properties of these fiber electrodes and their hydrogen peroxide (H_2_O_2_) sensing capability and showcase their applications *in vivo* (rodent) and *ex vivo* (bladder tissue). This scalable process fabricates high-performance microfiber electrodes that can be easily customized and deployed in diverse bioelectronic monitoring and stimulation studies, contributing to a deeper understanding of health and disease.

## Introduction

Biological signal transduction and cellular communication rely on a plethora of electrical, electrochemical, and chemical processes.^1^ Disruptions in these pathways can directly result in physiological disorders. Bidirectional interfaces with tunable electrical, chemical, microfluidic, and optical properties have enabled investigations into and modulation of complex pathways.^2,3^ Conventional bioelectronic interfaces rely on highly conductive noble metals, such as Au and Pt, and semiconductors (Si). However, though these electrodes (e.g., Utah array) have achieved small dimensions and dense electrode packing, they are expensive, rigid, and have a limited surface area and high impedance, leading to a lower signal-to-noise ratio (SNR) and charge transfer properties for stimulation.^4,5^ Numerous materials have been investigated for developing bidirectional bioelectronic interfaces, most notably carbon-based (e.g., carbon fiber,^6^ CNT,^7^ graphene^8^) and conductive polymers.^9^ Carbon-based electrodes are soft, lightweight, and—in addition to recording and stimulation—can be used for sensing neurotransmitters like dopamine.^10−12^ Carbon fibers, however, are brittle: they require a millimeter-scale shell for support during electrode insertion, which limits penetration depth and increases the overall device footprint.^13^ They also lack electrocatalytic capabilities that limit the sensitivity for detecting certain chemical species like hydrogen peroxide (H_2_O_2_), which is important in studying degenerative diseases like Alzheimer’s and Parkinson’s.^14^ Conductive polymers such as poly(pyrrole) (PPy), poly(aniline) (PANI), and poly(3,4-ethylene dioxythiophene) (PEDOT) face impediments due to trade-offs between electrical conductivity and processability, and issues regarding long-term stability and delamination.^15^ Therefore, it is important to identify materials with high conductivity, biocompatibility, and the required mechanical properties.

MXenes have the potential to address the aforementioned material and processing challenges. MXenes are a large family of layered two-dimensional (2D) transition metal carbides, nitrides, and carbon nitrides, offering the highest electrical conductivity among solution-processable 2D materials.^16,17^ The most optimized MXene, Ti_3_C_2_T_*x*_, can achieve an electrical conductivity of over 20 000 S/cm, approximately 5–10 times higher than reduced graphene oxide (rGO).^18^ Their 2D morphology, easy processability, and hydrophilic functional-group-decorated surfaces make them ideal coating materials for various substrates, offering functional enhancements with minimal increase in weight. Since their discovery at Drexel University in 2011, MXenes have achieved repeatable kilogram-scale productions and have experienced rapid growth across various fields, particularly in biomedical applications.^19−21^ These span from electrophysiology, biosensing, tissue engineering, and therapeutics to antibacterial and antiviral applications.^22−24^ Specifically, Ti_3_C_2_T_*x*_ MXene is biocompatible and does not exhibit cytotoxicity to a wide variety of mammalian cells and tissues.^25−29^ It has enabled implantable and wearable bioelectronics for invasive and noninvasive recording of animal and human electrophysiology.^27,30−32^ Ti_3_C_2_T_*x*_ MXene microelectrodes displayed a 4-fold lower impedance and greater capacitance than gold electrodes, leading to enhanced electrophysiological sensing capabilities with a higher SNR.^27^ Moreover, MXene electrodes can be conveniently sterilized using conventional methods without chemical and performance degradation.^33^ Despite the impressive performance demonstrated shown by earlier MXene electrodes, challenges persist in tissue insertion due to their planar and thin geometry, limited versatility issues due to prefixed designs, suboptimal MXene conductivity caused by the misalignment of MXene flakes, and the time- and resource-intensive nature of microfabrication. Electrical conductivity aside, Ti_3_C_2_T_*x*_ MXene is highly sensitive to H_2_O_2_, as it exhibits a low reduction onset potential due to its electrocatalytic capabilities.^34^ However, this capability has not been demonstrated without a bulky current collector or within biological systems.^35^ Therefore, the demand exists for miniaturized MXene electrodes with simplified fabrication, high electrical conductivity, and integrated capabilities for electrical and chemical sensing, as well as modulation.

Compared to electrodes of other geometries, fiber-shaped electrodes provide higher spatial and temporal resolution because of their proximity to target biological cells.^36^ A small electrode diameter (*D*) decreases insertion resistance and associated damage. It significantly reduces the bending stiffness *K* ∝ *D*^4^, resulting in less tissue damage and signal loss—vital for long-term implants.^37^ Furthermore, unlike planar electrodes with fixed device configurations, microfiber electrodes offer greater flexibility and fewer geometric constraints. They can be inserted into or wrapped around target tissues of varying geometries, even positioned between cells as individual electrodes or as a bundle.^38−40^ This versatility makes them applicable to various scenarios. Recently, thermal drawing has emerged as a powerful method for fabricating versatile neural probes.^41,42^ However, this process imposes limitations on material selection and faces challenges in reducing the diameter below 100 μm. Additionally, the slow production speed makes thermal drawing challenging for meeting the large-scale and multimodal requirements of biointerfaces. The easy processability and multifunctionality of MXenes provide an alternative, streamlined process for making high-performance, fiber-shaped electrodes.

Here, we propose a rapid, scalable, and versatile dip-coating technique for manufacturing electrically conductive and flexible Ti_3_C_2_T_*x*_ microfiber electrodes that can be easily customized for various biological studies. This method produces MXene-coated fibers with tunable mechanical, electrical, and electrochemical properties, featuring uniform MXene coatings that are precise and reproducible. These qualities were unattainable in previous MXene fiber coating studies.^43,44^ Moreover, we utilized the shear force in the MXene solution meniscus during dip coating to align and conformally coat MXene flakes along the surface of individual nylon filaments, a strategy that has not yet been utilized in the coating of MXene fibers. This achieved low linear resistance (as low as 9.3 ± 1.1 Ω/cm) even at high drawing speeds (up to 15 mm/s). These MXene-coated filaments can be efficiently batch-fabricated into arrays of fiber electrodes, encapsulated with an insulating layer of Parylene C, and exposed only at the tip upon application for electrical recording, stimulation, and H_2_O_2_ sensing. The MXene fiber microelectrodes exhibited excellent knotting and favorable mechanical properties for handling and implantation. We demonstrated the versatility of these simply made, multifunctional fiber electrodes both *in vivo* in rat nerves and muscles and *ex vivo* in bladder tissue. These fiber microelectrodes provide an economical, durable, and user-friendly miniaturized platform for various biological applications.

## Results and Discussion

### MXene-Enabled Microfiber Electrode Fabrication

In this study, we leveraged the advantageous characteristics of MXenes and nylon to fabricate flexible, fiber-shaped microelectrodes with dip coating. Nylon filaments were chosen as substrates due to their lightweight, high chemical resistance, mechanical durability, and cost-effectiveness.^45^ Moreover, it is a U.S. Food and Drug Administration (FDA) approved material for medical devices such as permanent sutures, catheters, and dental implants.^46,47^ The nylon fibers are produced through melt spinning in a continuous manner. The positive charge on nylon leads to strong binding of the negatively charged MXene.^43^ If a continuous MXene coating is applied, it can enable electron conduction along the entire length of the filament. Round nylon filaments of varying diameters are chosen for this proof-of-concept study to simplify MXene/substrate interactions in the dynamic meniscus and ensure comparability with current fiber microelectrode probes in bioelectronics.

**Figure 1:**
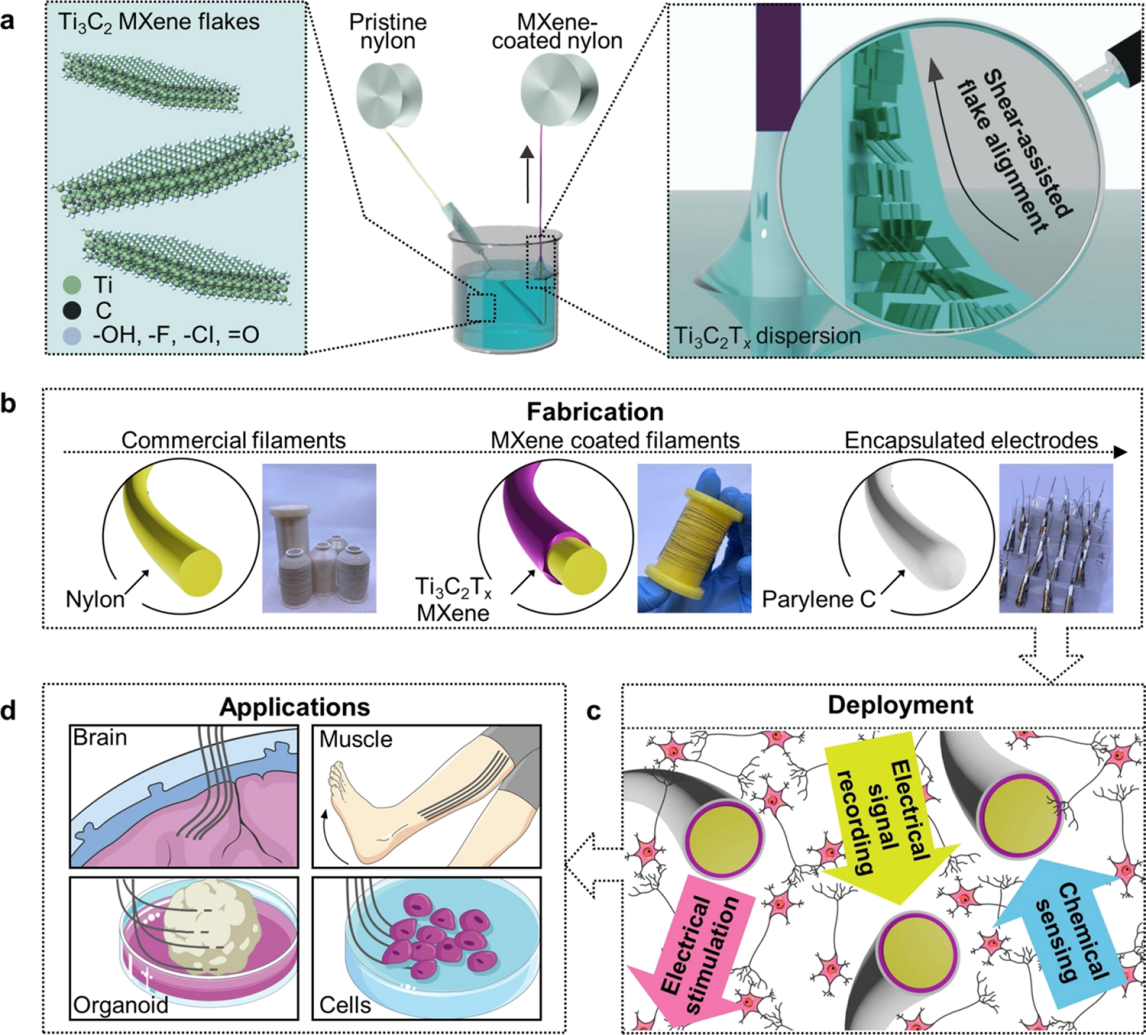
Schematics of the fiber electrode fabrication and potential applications. (a) Schematic of the dip coating process consisting of a nylon filament running through a dispersion of single-layer MXene flakes. During this process, MXene flakes are aligned in the axial direction by the shear force present in the meniscus. (b) Schematics and digital photographs of the nylon filaments, MXene-coated nylon filaments, and Parylene-coated electrodes. (c) Schematic of cut electrodes with the cross-section of the MXene coating exposed and their demonstrated capabilities in this work. (d) Potential applications of the MXene electrodes in various biological systems, including cells, organoids, brains, and muscles.

Single-layer Ti_3_C_2_T_*x*_ flakes, with an average flake size of 1 μm, were synthesized through a mixed-acid method. The successful synthesis was confirmed using UV–vis spectroscopy and X-ray diffraction (XRD) analysis (Figure S1).^20,48^ The abundance of negatively charged functional groups (e.g., –F, −CI, OH) on Ti_3_C_2_T_*x*_ flakes lead to a large negative ζ-potential in water (−50 mV), allowing them to form a stable solution for dip coating without any surfactants or additives. Furthermore, the solution rheology can be tuned over a wide range by adjusting MXene flake size and/or concentration, eliminating the necessity for rheological thinners and thickeners. The simple coating formulation of MXene in water is preferable for implantable electrodes when additives may complicate the biological response. In contrast to graphene oxide (GO), MXene does not require thermal or chemical reduction to achieve high conductivity. The MXene dip coating process is illustrated in Figure 1a: the nylon filament is immersed in the MXene solution and vertically drawn. A stable meniscus forms thanks to the favorable electrostatic interactions between nylon and MXene. This enables a consistent layer of MXene dispersion to adhere to the fiber, where MXene flakes are aligned by shear force. This layer dries into a MXene coating at a uniform thickness (Movie S1).

MXene-coated filaments can be selected based on their properties, cut into desired lengths for intended applications, and encapsulated with a 10 μm thick layer of Parylene C (Figure 1b). Parylene is a United States Pharmacopeia (USP) Class VI polymer that is widely used as a biocompatible encapsulation for chronic medical devices.^49,50^ It has become a popular substrate and passivation layer for bioelectronics due to its barrier properties, flexibility, and processability.^49^ Once the encapsulated fiber is cut at the tip, a controllable amount of MXene is exposed, providing the benefits of easy handling and reproducible outcomes (Figure 1c). This represents a significantly simplified electrode fabrication and deployment process compared to microfiber electrodes made from other materials, e.g., brittle carbon fibers that require extensive polishing.^37,51,52^ These electrodes can be produced to different lengths to investigate biological systems at various scales, ranging from cells (∼10–20 μm), organoids (∼1–5 mm), and tissues (>1 cm) to the brain and muscles, *in vivo*, *in vitro* or *ex vivo* (Figure 1d).

### Tailoring Electrode Properties Through Dip Coating Parameters

Though dip coating is a widely used technique for applying MXenes to various substrates like glass, PET, foams, yarns, and fabrics owing to its simplicity and control, this method has yet to be demonstrated in a continuous fashion on a regularly shaped single filament.^18,53^ Fluid dynamics theories, most notably the Landau–Levich–Derjaguin (LLD) model, have been developed to describe the flow dynamics within the meniscus and bulk solution when a fiber is vertically withdrawn from the surface of a Newtonian liquid. This process involves an equilibrium between the capillary action induced by substrate drawing and the viscous drag exerted by the liquid.^54^ The coating thickness (*h*_fiber_) on fine fibers can be approximated as follows
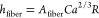
1where *A*_fiber_ is 1.34, a unitless value representing the effect of the fiber geometry on the interface; *R* is the fiber radius; capillary number (*Ca*) is defined as *U*η/σ, where *U* is the drawing speed, η is viscosity and σ is the liquid’s surface tension. Thus, an increase in drawing speed, viscosity, and fiber diameter should increase coating thickness. However, the model becomes considerably more complicated for non-Newtonian fluids. MXenes suspensions exhibit shear thinning behavior, where their viscosity decreases with an increase in shear force, the extent of which depends on the size distribution of MXene flakes and their concentrations.^55,56^

Additionally, due to their large aspect ratio, MXenes can spontaneously organize into a liquid crystalline state above a critical transition concentration, which can be calculated for a given suspension based on the MXene flake size and concentration.^57^ The liquid-crystalline state of MXenes has been to facilitate flake alignment during wet spinning or blade coating, where shear force is present.^57,58^ Consequently, during dip coating, the shear force distribution gradients in the meniscus will be influencing and be influenced by the local viscosity and alignment of MXene in solution, making the theoretical prediction of MXene coating thickness challenging.^59^ Thus, we designed an experimental approach to investigate the impact of three parameters—namely MXene concentration, filament diameter, and drawing speed—on coating thickness and resulting properties.

**Figure 2:**
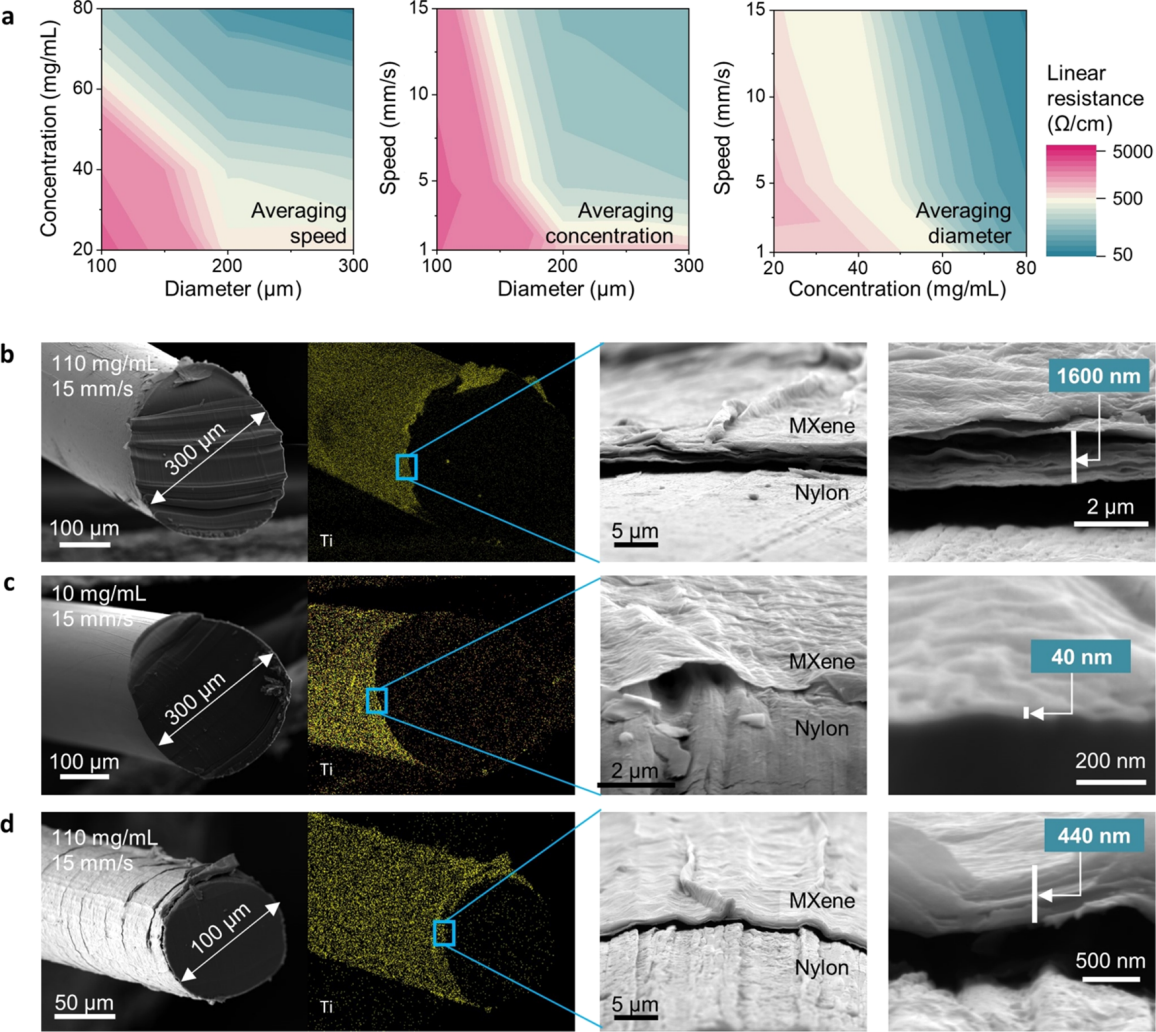
Tunability of the facile filament dip coating process. (a) Parametric study of linear resistance with the filament diameter, MXene concentration, and drawing speed. Cross-sectional SEM images and EDS maps of 300 μm nylon filament coated using (b) 110 and (c) 10 mg/mL MXene solution, as well as (d) 100 μm filament coated using 110 mg/mL MXene solution. Samples were coated at 15 mm/s drawing speed. EDS images are in the net count with brightness increases of 60, 90, and 60%, respectively.

A parametric study was conducted across three levels for each parameter: 20, 40, and 80 mg/mL for MXene concentration; 100, 200, and 300 μm for nylon filament diameter; and 1, 5, and 15 mm/s for coating speed. The maximum speed allowed for the dip coating setup is 15 mm/s. Increasing the MXene concentration significantly raises the viscosity of the solution, transforming it from a liquid resembling water to a paste, despite shear thinning being observed across all employed MXene concentrations (Figure S2). Consistent and uniform coatings were achieved on all 27 conditions (*n* = 5 samples per condition). The resistance measurements of the coated filaments were summarized (Figure 2a and Table S1). As MXene concentration, filament diameter, and drawing speed increased, the resistance decreased by more than 2 orders of magnitude, from over 5 kΩ/cm to below 40 Ω/cm. This aligns with the trends predicted by eq 1, indicating the presence of comparable fluidic dynamic mechanisms within the explored parameter space. Furthermore, standard deviation (SD) decreased with average resistance, indicating a more uniform coverage and enhanced electron percolation network with the accumulation of MXene flakes (Figure S3).

Additionally, to ascertain the relative impact of each parameter, we presented the mean resistance for each parameter in a main factor analysis fashion, which revealed that MXene concentration and filament diameter exert a more substantial influence (Figures S4 and S5). Therefore, the range of MXene concentration and filament diameter was further expanded by adding concentrations of 10 and 110 mg/mL and filaments with a diameter of 28 μm. A concentration of 110 mg/mL was the highest concentration we achieved through a centrifugation-based concentration method. The trend of decreasing resistance with increasing concentration persisted, with the 110 mg/mL MXene solution yielding the lowest linear resistance in this study, measured on the 300 μm filament (Figure S6). Similarly, the trend of increasing resistance with decreasing diameter continued for 28 μm filaments, producing a coating with a resistivity of ∼600 Ω/cm, when the 110 mg/mL MXene solution was used.

To gain insight into the coating mechanism, we utilized scanning electron microscopy (SEM) imaging to examine the MXene coatings on specific filaments (Figures 2b–d and S7). The energy dispersive spectroscopy (EDS) confirmed the conformal MXene coatings on the nylon fibers. For a 300 μm-diameter filament, the 110 mg/mL MXene solution produced a thick coating, measuring approximately 1.6 μm in thickness, 20 μg/cm in weight with a metallic purple hue and a slightly wrinkled morphology characteristic of Ti_3_C_2_T_*x*_ (Figure S8). In contrast, the 10 mg/mL MXene solution resulted in an ultrathin coating of approximately 40 nm with negligible weight increase. The 11 times difference in MXene concentrations brought a 40 times difference in coating thickness. Based on eq 1, the disproportional difference can be attributed to the significantly higher viscosity of the 110 mg/mL dispersion than 10 mg/mL. The thickness of the 110 mg/mL coating, measuring 1.6 μm, is notable, given that low MXene concentrations (1–30 mg/mL) are typically recommended and employed in dip coating.^43,44,60−63^ These dilute MXene solutions lead to thin MXene coatings with high resistances, requiring the repetition of dip coating cycles to achieve a thicker deposition. In our study, we demonstrated that viscous MXene solutions, reaching up to 110 mg/mL, are suitable for dip coating when assisted by the high shear force generated from fast coating speed. This enabled the rapid and continuous production of highly conductive filaments in a single pass, with low MXene consumption (<20 μg/cm) resulting in minimal coating costs. Without the use of temperature evaluation and specialized equipment, and the accompanying substrate material limits, as in the case of thermal drawing,^64^ this method is more cost-effective and accessible for production purposes. On the effect of filament diameter, at the same concentration of the 110 mg/mL solution, the filament of a smaller 100 μm diameter received a thinner coating of 440 nm, as anticipated from eq 1.

When examining resistance in conjunction with coating thickness, an intriguing observation emerges. The 40-fold difference in coating thickness between 110 and 10 mg/mL of MXene on 300 μm diameter filaments resulted in a 73-fold decrease in resistance (Table S2). If we approximate MXene coating as a free-standing film and calculate electrical conductivity, the coating from the 110 mg/mL MXene solution should have a conductivity of 7093 ± 819 S/cm, while the coating from the 10 mg/mL MXene solution should have a conductivity value of 3891 ± 249 S/cm. The former approaches the electrical conductivity of 8875 ± 412 S/cm measured for a free-standing film created by vacuum-assisted filtering of the same Ti_3_C_2_T_*x*_ solution. We attribute the higher conductivity from 110 mg/mL to its liquid crystalline ordering and its resulting higher order of flake alignment in the presence of shear force in the meniscus, which is confirmed by rocking curve XRD analyses of the (002) peaks of the coatings (Figures S9 and S10). This higher order of alignment allows the 110 mg/mL MXene-coated filaments to demonstrate not only lower resistances than commercial silver-plated nylon filaments but also at a lower amount (wt %) of active material (Figure S11 and Table S3). For example, 100 μm nylon filaments coated with 110 mg/mL MXene exhibit a resistance of 41.9 ± 6.1 Ω/cm at 5.3 wt % active material loading, contrasting with the 200 Ω/cm resistance at a 10.0 wt % for silver-plated nylon of the same diameter. Low linear resistance is critical to the performance of fiber-shaped electrodes, as the overall electrode resistance scales linearly with fiber length. It implies that MXene fiber electrodes will not require a current collector, eliminating the need for additional steps such as gold deposition^65^ and metal wire attachment^66^ that are typically required to enhance the electrical conductivity of rGO electrodes.

We then calculated the bending stiffness of MXene-coated nylon electrodes, a critical property influencing the mechanical compatibility of the electrodes with tissues (Table S4).^36^ Rigid electrodes are less compatible with soft tissues since they trigger increased stiffness-related foreign body response.^67^ The subsequent immune response and device encapsulation reduce the recording and stimulation capabilities. Thus, the lower stiffness of MXene-coated and Parylene-encapsulated electrodes compared to other conductive fibers that have been considered as neural probes, such as carbon fiber and fibers made of CNT, platinum, tungsten, and silicon at comparable diameters, is beneficial (Figure S12). This suggests that MXene electrodes may possess greater mechanical compatibility with biological tissues, such as brains and muscles. Despite their lower stiffness, their fiber geometry allowed direct, assistance-free insertion of all fibers (28–300 μm diameter) directly into deep tissue, modeled with 0.6 wt % agarose gel—a composition commonly employed to mimic the mechanical properties of the brain (Figure S13).

By varying processing parameters, we demonstrated dip coating as a facile method for producing conductive filaments with tunable MXene coating thickness, mechanical and electrical properties. For the subsequent electrochemical characterizations, meters of coated filaments with varying parameters were produced quickly and cut into 5 cm-long electrodes and then encapsulated with Parylene C. Thanks to the low resistance from aligned MXene flakes, these electrodes can be made into longer lengths for deep brain or muscle stimulation in large animals (e.g., nonhuman primates^68^) without concerns about signal loss associated with a significantly higher resistance. These MXene electrodes can be easily “activated” for deployment and shortened, if necessary, by cutting them with a fresh razor blade against a hard surface (e.g., a glass substrate) at room temperature. This method is adequate for producing regular and consistent cross sections (Figure S14) for electrochemical characterization, which was performed to assess the performance, reliability, and stability of the MXene electrodes.

### Electrochemical Characterization of MXene Electrodes

While low impedance and high capacitance are typically desirable for effective sensing and stimulation, the specific physical, mechanical, and electrochemical requirements for electrodes vary depending on the targets and goals. For example, clinical deep brain stimulation platforms necessitate electrode geometries with a high aspect ratio.^69^ In contrast, transcutaneous electrical nerve stimulation requires larger electrodes capable of delivering higher magnitudes of stimulation currents.^70^ This renders our method especially valuable for numerous biological applications. To ensure proper electrode selection and safe operation, it is essential to conduct electrochemical characterizations on MXene dip-coated electrodes produced under various processing parameters. In addition, to ensure the success of electrode application, the electrodes should exhibit high reliability and stability. Cyclic voltammetry (CV) and electrochemical impedance spectroscopy (EIS) tests were conducted to provide insights into the capacitance and impedance of MXene electrodes in relation to both MXene concentration and filament diameter in a three-electrode setup (Figure S15).

**Figure 3:**
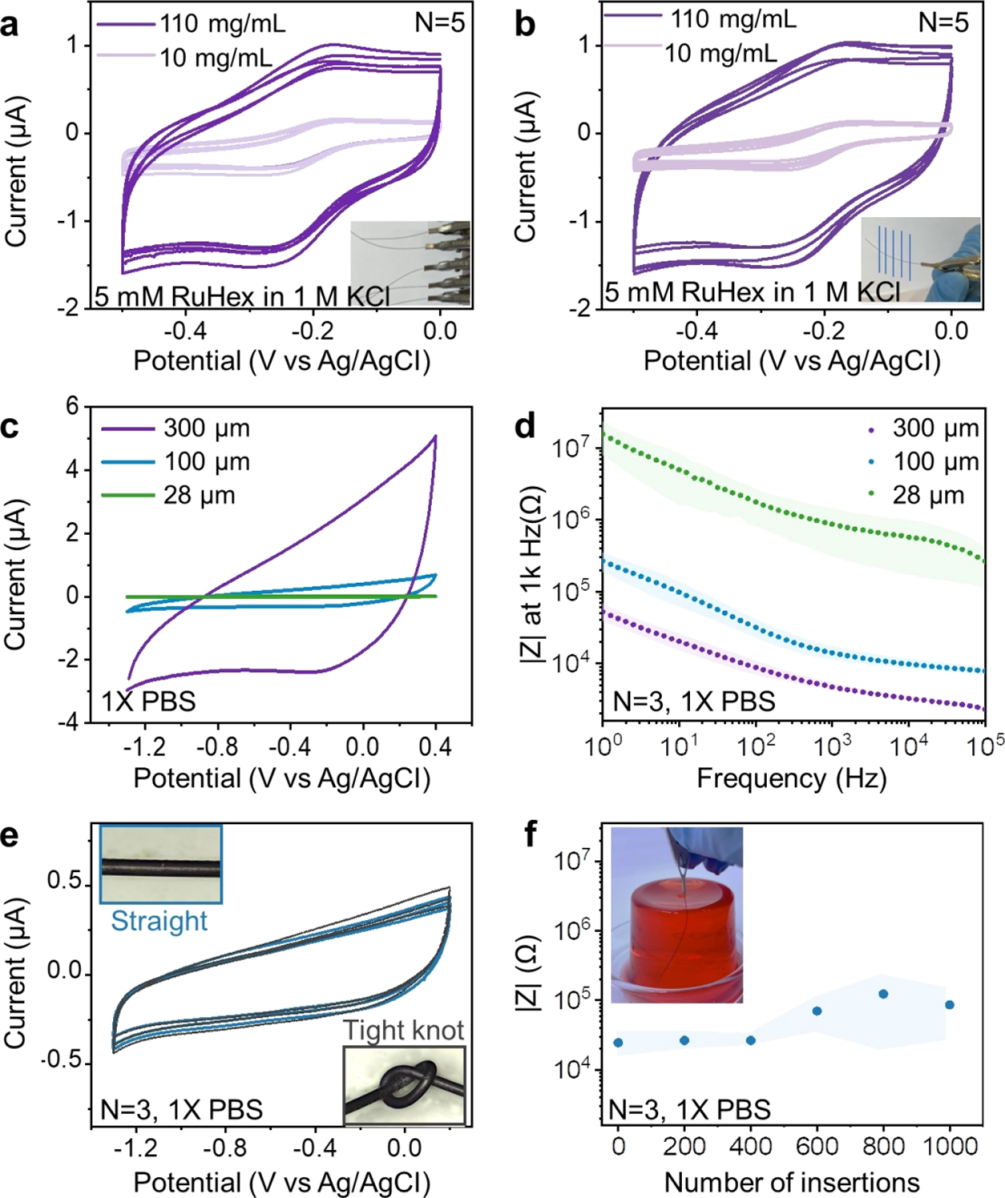
Electrode characterization in common electrolytes and analytes for biological applications. Cyclic voltammograms (CVs) of 300 μm electrodes as a function of MXene concentration (10 and 110 mg/mL) in 5 mM RuHex in 1 M KCl at 20 mV/s and reproducibility between (a) different electrodes and (b) multiple cuts of the same electrode. (c) CVs at 100 mV/s and (d) impedance modulus of 110 mg/mL MXene coated electrodes as a function of frequency for 3 different diameters (300, 100, and 28 μm) in 1× PBS. Data are plotted as average values with shaded regions corresponding to SDs. (e) CVs before and after knotting of 100 μm electrodes at 100 mV/s in PBS. (f) Average impedance and SD at 1 kHz in 1× PBS over 1000 insertions (100 μm electrodes coated with 110 mg/mL inserted to a depth of 3–4 cm) into 0.6 wt % agarose. The drawing speed for all tested electrodes is 15 cm/s.

First, 10 electrodes of 300 μm diameter, 5 coated with 10 mg/mL and the other 5 coated with 110 mg/mL MXene, were characterized with CV in 5 mM hexamine-ruthenium(III) [Ru(NH_3_)_6_]^3+^ (RuHex) in 1 M KCl at 20 mV/s (Figure 3a). RuHex, a standard outer-sphere redox probe, was chosen as the electrolyte due to its sensitivity to surface area (not sensitive to surface chemistry). The high degree of CV overlap among different electrodes indicates a consistent and uniform coating. A higher capacitance was observed when comparing electrodes coated with a concentration of 110 mg/mL to those coated with 10 mg/mL. This is attributed to the thicker coating resulting from the higher concentration of MXene in the 110 mg/mL solution. For the same reason, lower impedance was observed in the 110 mg/mL electrodes compared to the 10 mg/mL electrodes (Figure S16). Moreover, the oxidation and reduction of RuHex occurred at a centered potential of −0.2 V (vs Ag/AgCl), as expected.^71^ The semisigmoidal CV shape suggests minimal diffusion limitations, which can improve speed and sensitivity in analyte detection. Next, one electrode was chosen from each of the 110 and 10 mg/mL sets. A CV was recorded after an initial cut, and then the incision was retracted by 1–2 mm, exposing a fresh cross-section of the same fiber, followed by recording another CV. This process was repeated for a total of five iterations (Figure 3b). Consistent high degrees of overlapping CVs both support the uniformity of the coating and also underscore the reliability of the electrode-cutting method. This method consistently exposes uniform cross sections. The effectiveness of the Parylene C insulating layer is evident from the significantly higher capacitance and lower impedance observed in nonencapsulated electrodes compared to the encapsulated ones (Figure S17).

To understand the relationship between fiber diameter, capacitance, and impedance, we selected 110 mg/mL MXene-coated electrodes with diameters ranging from 300 to 28 μm. We employed 1× phosphate-buffered saline (PBS), a simplified representation of human body fluids commonly used in electrochemical characterizations of recording and stimulation electrodes. This choice allowed for a meaningful comparison between MXene electrodes and electrodes made from other materials. The safe operational window for MXene fiber electrodes in PBS, determined at a scan rate of 100 mV/s, ranges from −1.3 to 0.4 V, with 28 μm electrodes exhibiting a slightly earlier onset of oxygen evolution reaction (Figure S18). As the diameter decreased from 300 to 28 μm, a notable reduction in capacitance was observed due to the thinner coating and smaller exposed MXene ring at the electrode tips (Figure 3c). The cathodic charge storage capacity (CSCc) was determined from CV scans by integrating the cathodic current over time and normalizing it by both the entire cross-sectional area and the active material area, providing indicators for the sensing performance of the electrode and the active material (MXene), respectively. Larger CSCc values indicate higher sensitivity to electrical signals.

When compared with electrodes of other materials such as carbon fiber, graphene fibers, CNT, SI, PEDOT:PSS, and Pt, MXene electrodes exhibited significantly higher CSCc when normalized by the active area of the coating (Table S5). The heightened performance is likely attributed to two factors: the advantageous diffusion in microelectrodes due to the reduction in electrode diameter and coating thickness, and the increased surface area resulting from the 2D stacked morphology of MXene, facilitating capillary actions when the edges are exposed. This observation aligns with the results of a recent study that compared 2 μm thick MXene thin film electrodes with edge-exposed and basal-exposed configurations.^71^ These factors also provided the 300 and 100 μm electrodes with low impedances of 4.70 ± 0.55 and 14.0 ± 3.1 kΩ, respectively, at 1 kHz—among the lowest reported for implantable microfiber electrodes (Figure 3d and Table S5). The phase responses from EIS are typical for smooth, compact electrodes, consistent with the SEM and XRD findings for the MXene electrodes (Figure S19).^72^ Finally, we observed an increase in impedance and variations with decreasing fiber diameter due to the reduction in MXene surface area available for charge exchange, presenting challenges in recording high-quality electrical signals with 28 μm thin electrodes.

Furthermore, the MXene coating, nestled between the substrate polymer and the encapsulating layer of Parylene C, exhibited exceptional knotting performance. Identical CVs were recorded before and after tying knots as tightly as possible, representing a more mechanically demanding test than bending and knotting with less curvature (Figures 3e and S20). Electrode durability to insertion was also evaluated. Only a slight change in impedance was observed after 1000 repeated insertions, with negligible alteration in impedance and CV (Figures 3f and S21). Furthermore, while achieving long cyclability proved challenging within the extended window of −1.3 to 0.4 V in 1× PBS, reducing the window to −1.1 to 0.3 V obtained excellent cyclability of sustained performance over 5000 cycles at 100 mV/s by avoiding hydrogen and oxygen evolution reactions near the cathodic and anodic turnover potentials (Figure S22). Only minimal capacitance and impedance changes were observed even after 12 months of bench storage (Figure S23). This can be attributed to Parylene effectively protecting MXene against moisture in the air, a factor known to induce hydrolysis and oxidation of MXene, but also to the high stability of Ti_3_C_2_T_*x*_ once it is processed into a film.^73^ When the 12 months electrode was immersed 35 mm versus 2 mm depth into 1× PBS, a consistent performance was observed, allowing for reliable readings from different depths within a tissue (Figure S23). Collectively, all tests suggest the versatility of the MXene dip coating process in reliably producing robust, fiber-shaped electrodes with varying capacitance and impedance.

### *Ex Vivo* Sensing of H_2_O_2_ in Murine Bladder Urothelium

Reactive oxygen species (ROS) are essential for cellular processes, including signaling, immune responses, and redox regulation. Their irregularities induce oxidative stress, causing significant biological damage, leading to chronic diseases, acute conditions, and aging.^74^ One of the most important ROS is H_2_O_2_, the fluctuation of which serves as an indicator for Alzheimer’s and Parkinson’s diseases.^35^ Thus, measuring and monitoring H_2_O_2_ is key for monitoring oxidative stress and assessing the impact of ROS in cellular and physiological processes.

**Figure 4:**
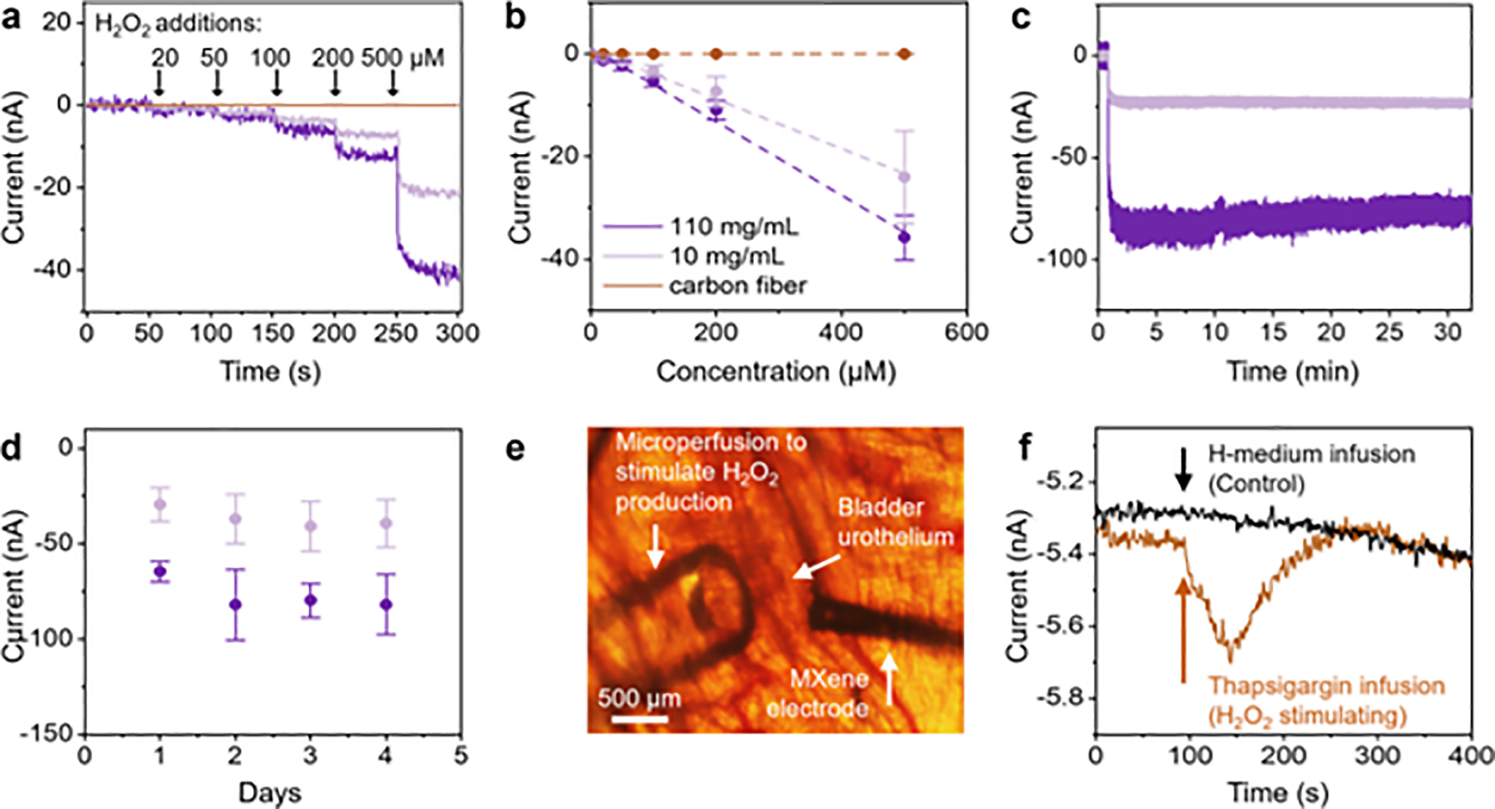
H_2_O_2_ sensing and *ex vivo* demonstration in bladder urothelium. (a) Amperometry at −650 mV (vs Ag/AgCl) with additions of H_2_O_2_ (20, 50, 100, 200, and 500 μM) every 50 s. The electrodes are 300 μm electrodes coated with 10 mg/mL MXene and 110 mg/mL MXene, as well as carbon fiber electrodes. (b) Calibration curve of H_2_O_2_. (c) Stability of response signal during 30 min–1 mM of H_2_O_2_ at *t* = 50 s using amperometry. (d) Stability of response to 1 mM H_2_O_2_ over days. (a–d) Share the same legend. (e) Optical microscopy image of the bladder urothelium, where a 10 mg/mL MXene fiber microelectrode was placed in contact of the urothelium, which was constantly perfused with H-medium. Peroxide production was stimulated with 200 nM thapsigargin using microperfusion. (f) Amperometric detection of peroxide using MXene fiber microelectrode at −650 mV vs Ag/AgCl. The arrow indicates the point at which either H-medium (black trace) or 200 nM thapsigargin (brown trace) was microperfused onto the urothelium.

We employed chronoamperometry to reduce H_2_O_2_ at −650 mV (vs Ag/AgCl), considering the limited positive potential window for H_2_O_2_ oxidation determined in Figure 3c, for two types of MXene electrodes: 300 μm electrodes coated with 10 and 110 mg/mL MXene, respectively. H_2_O_2_ concentrations (20, 50, 100, 200, and 500 μM) were sequentially added every 50 s to 10× PBS (Figure 4a). The selection of these two types of electrodes allowed us to understand the effect of MXene coating thickness (exposed area of MXene at the tip) on the sensitivity of H_2_O_2_ detection. We observed a good linear response in current for both 10 mg/mL (*r*^2^ = 0.810) and 110 mg/mL (*r*^2^ = 0.965) MXene-coated electrodes across the entire concentration range (Figure 4b). While the slope of the line, representing the sensitivity of the whole electrode, was higher for 110 mg/mL, normalization by the cross-sectional MXene area demonstrates that the 10 mg/mL electrode had a higher sensitivity (−148.39 μA/μM/cm^2^) compared to the sensitivity of 110 mg/mL electrode (−4.73 μA/μM/cm^2^) per unit area of the active material. This is likely attributed to enhanced diffusion resulting from the decrease in coating thickness (Figure S24). The electrodes exhibited excellent stability in continuous runs (lasting 30 min with 1 mM of H_2_O_2_) and over consecutive days with 1 mM of H_2_O_2_ (Figure 4c,d). In comparison, carbon fiber electrodes, while exhibiting the usual electrochemical performance against RuHex (Figure S25), showed no response toward H_2_O_2_ due to the lack of electrocatalytic activity. These results underscore the H_2_O_2_ detection capability of the MXene-coated electrodes, all without the need for any additional current collector or chemical treatment. This achievement is attributed to the well-aligned flakes during coating and the redox-active surface of MXene flakes.

Next, we utilized a 110 mg/mL MXene-coated electrode to monitor H_2_O_2_ production in bladder urothelium when stimulated using 200 nM thapsigargin through microperfusion (Figure 4e). This endeavor led to the successful detection of H_2_O_2_ amperometrically from bladder urothelium tissue. As shown in Figure 4f, upon perfusion of 200 nM thapsigargin, a larger current was observed and attributed to the production of H_2_O_2_. Our study proves the capability of MXene for H_2_O_2_ sensing in a biological system, enabling the translation of these findings into microfiber applications.

### *In Vivo* Electrical Recording and Stimulation in Sciatic Nerve

Electrical stimulation is one of the most prominent clinical techniques for modulating electrophysiological activity. Stimulation is achieved by injecting current pulses at the electrode-tissue interface, which induces depolarization of the cell membrane.^72^ This has enabled therapeutic interventions such as deep brain stimulation for epilepsy and transcutaneous electrical nerve stimulation for pain relief.^75,76^ The electrical stimulation performance of an electrode is typically assessed using chronopotentiometry, where a charge is injected through biphasic, charge-balanced current pulses into the electrode in a 1× PBS electrolyte.^9,72^ A cathodic current pulse results in the generation of a potential transient, with the cathodic excursion potential (*E*_c_) recorded 10 μs after the cathodic pulse concludes. For safe operation, the maximum charge of an electrode is determined as the charge injected when *E*_c_ reaches the cathodic limit established by CVs, which is −1.3 V, in the case of MXene electrodes. To facilitate direct comparisons across fiber electrodes of different sizes and materials, cathodic charge injection capacity (CIC_c_) is adopted as a standardized measure. This involves normalizing the maximum cathodic charge by area, either considering the entire cross-sectional area or the active material area.

**Figure 5:**
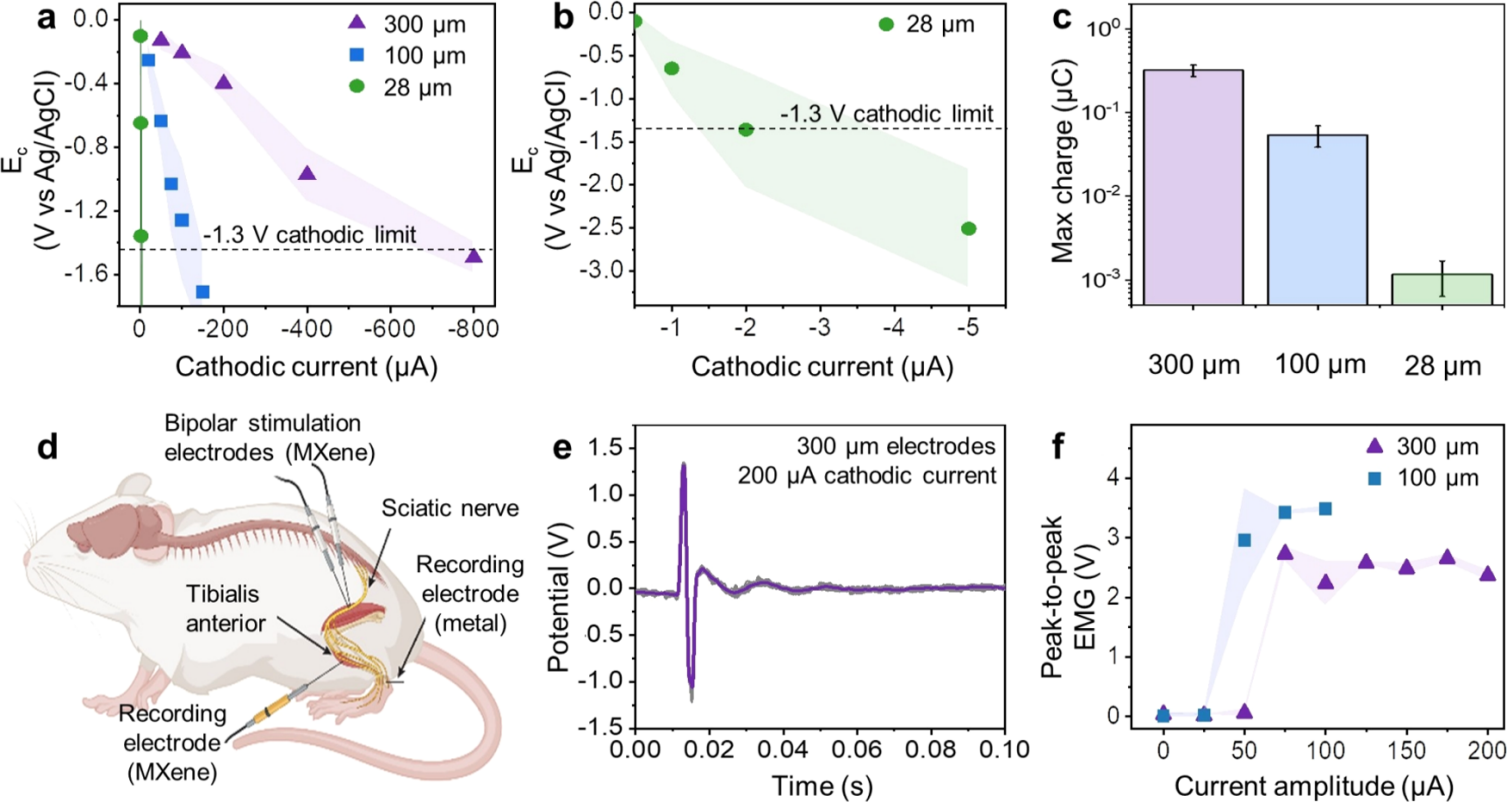
MXene-nylon microfiber electrodes can record and stimulate electrophysiological activity. (a) The cathodic potential excursion *E*_c_ values plotted against injected cathodic current amplitude for 110 mg/mL MXene, 15 mm/s coated electrodes with diameters of 300, 100, and 28 μm. The cathodic voltage limit of MXene electrodes is displayed as a dashed line and SD as shadows (*n* = 3). (b) The same plot on a scaled *X*-axis for 28 μm diameter electrodes. (c) The maximum charge injected (C) across electrodes of 3 different sizes. (d) Schematic of the *in vivo* stimulation and recording experiment with a pair of MXene electrodes placed on the sciatic nerve as the bipolar stimulation electrodes and another MXene electrode on the surface of the tibialis anterior muscle as the recording electrode. (e) Representative evoked electromyography (EMG) potential at the tibialis anterior as recorded by a 300 μm MXene electrode when the sciatic nerve is stimulated with a pair of 300 μm MXene electrodes at a cathodic current of 200 μA. Gray plots denote the individual pulses (*n* = 10), and the purple plots denote the average of individual traces. (f) Peak-to-peak evoked EMG as a function of stimulating current amplitude measured using 100 and 300 μm MXene microelectrodes.

In this study, we conducted chronopotentiometry on MXene electrodes with varying diameters (300, 100, and 28 μm). All electrodes were coated with 110 mg/mL MXene at 15 mm/s, with a set of 3 electrodes being prepared per size (Figures S26 and S27). We depicted the relationship between cathodic potential excursion *E*_c_ and increasing injected cathodic current amplitude, revealing the average *E*_c_ decrease with the reduction in electrode diameter (Figure 5a,b). The currents corresponding to *E*_mc_ at the cathodic limit were calculated as 642.2 ± 101.2, 108.2 ± 30.9, and 2.32 ± 1.05 μA, translating to maximum cathodic charges of 321.1 ± 50.6, 54.1 ± 15.4, and 1.16 ± 0.53 nC, respectively, as the electrode diameter decreased (Figure 5c). It indicates that thicker electrodes can withstand larger currents, enabling them to deliver more charge to the target tissue or cell if required to elicit a response. Consistent with the established scaling of CIC_c_ with electrode area, the 100 μm electrodes exhibit a higher CIC_c_ of 39.0 ± 11.1 mC/cm^2^ compared to the 300 μm electrodes at 18.8 ± 3.0 mC/cm^2^ (Figure S28).^31^ This is attributed to the fact that smaller electrodes facilitate faster ion diffusion and are less prone to nonuniform current distribution, which is referred to as edge effects. Comparing MXene electrodes with those made of other materials, the CIC_c_ of MXene electrodes is notably higher (Table S5). This can be attributed to the ring-shaped active area, which again mitigates nonuniform current distribution issues in round cross sections. Additionally, the slits in stacked MXene flakes likely facilitate the wicking of the electrolyte, thereby enlarging the actual surface area in contact with the electrolyte.

We then assessed the performance of our MXene fiber microelectrodes for *in vivo* electrophysiology studies. Here, we placed a bipolar assembly of our MXene fiber electrodes on the sciatic nerve of a rat for delivering stimulation current pulses (Figures 5d and S29). An independent MXene fiber electrode was placed on top of the tibialis anterior muscle to record the evoked electromyographic (EMG) activity. Upon application of a stimulating current pulse of 200 μA through a pair of 300 μm MXene electrodes on the sciatic nerve, evoked EMG was successfully recorded (Figures 5e and S30–S32). The SNR of the evoked EMG, as recorded by the MXene electrodes, was 15.20 ± 0.32 and 13.92 ± 0.53 dB for 100 and 300 μm MXene microfibers, respectively. Increasing the amplitude of the stimulation current on the sciatic nerve led to an increase in the peak-to-peak amplitude of the evoked EMG until maximum recruitment is achieved (Figure 5e). We note that maximum recruitment was approximately observed with 50 and 75 μA current pulses for 100 and 300 μm MXene fiber electrodes, respectively. This was achieved at current amplitudes much lower than the maximum amplitudes allowed or 100 and 300 μm MXene electrodes (642.2 ± 101.2 and 108.2 ± 30.9 μA, respectively). We benchmarked the stimulation and recording capabilities of the MXene microfibers against commercially available tungsten (W) and stainless-steel electrodes, respectively. Stimulation through the bipolar W electrodes follows a similar trend as that of the MXene electrodes, with maximum recruitment occurring around 80 μA (Figure S32). On the other hand, the evoked EMG recordings via the stainless-steel electrodes exhibited an SNR of 19.72 ± 0.52 dB (Figure S33). The greater SNR of these electrodes can be attributed to their higher conductivity and greater geometric footprint of these electrodes, since the lateral surface of the stainless-steel wires is exposed to the muscle tissue as well. This provides *in vivo* demonstration of MXene fiber microelectrodes for sensing and modulating electrophysiological activities.

**Figure 6:**
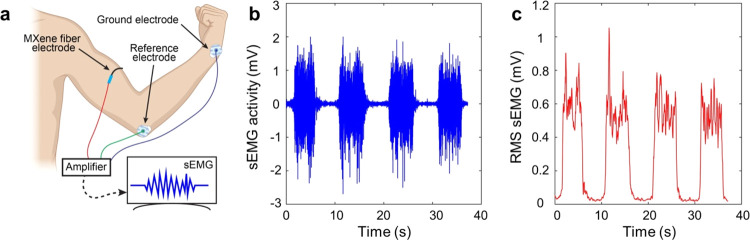
Surface electromyography (sEMG) recordings using MXene-coated nylon fiber. (a) Schematic illustrating the recording of sEMG activity during contraction of the biceps using a representative MXene-coated nylon fiber. (b) sEMG activity and (c) root-mean-square of the sEMG activity recorded at the biceps using a 300 μm diameter MXene-coated nylon fiber electrode. The marked black lines denote muscle contractions.

Furthermore, we tested the potential of our MXene-coated fiber microelectrodes for wearable and clinical applications. We placed a representative 300 μm diameter MXene-coated nylon fiber (without Parylene C encapsulation, 80 mg/mL MXene coated at 15 mm/s) on the skin over the belly of the biceps muscle of a healthy volunteer (Figure 6a). The microelectrodes were able to successfully record sEMG activity during voluntary contraction of the biceps (Figure 6b,c). This demonstrates the potential of our MXene fibers to be integrated into textiles as wearable sensors, further showcasing the versatility of microfiber electrode applications.

Through this study, we demonstrate dip coating as an efficient and versatile method for producing MXene-functionalized filaments with customizable properties. The continuous dip coating process shows promise for seamless integration into nylon filament production, ensuring uninterrupted electron conduction throughout the filament’s length. It would be interesting to apply this method to filaments of other materials, such as biodegradable polylactic acid and carbon fibers, as well as filaments of alternative geometries like hollow or grooved shapes. Hollow filaments can add microfluidic functions, while the grooved filaments have more surface for MXene to adhere. Various MXene compositions can be adopted (Figure S34), including V_2_CT_*x*_, which has demonstrated improved performance for ROS sensing, and Ti_3_C_2_T_*x*_/GO, where GO enhances sensitivity to numerous neurotransmitters.^11,77^ More parametric studies will be needed to understand how changes to other fiber substrates and MXene formulations, as well as how substrate chemistry, tension, and geometry interact with MXene flake size, concentration, and composition. These interactions will determine the optimal combination of coating parameters for conductivity and productivity. It is likely that fibers with delicate surface structures are best coated with MXene dispersions of low viscosity to penetrate small gaps and achieve highly conformal coatings. It is essential, in the next step, to construct a new coating setup that enables continuous automated production and accommodates faster drawing speeds. This will allow for quick additional parametric studies and experimental verification of the highest drawing speed for a given substrate and MXene dispersion.^78,79^ This adjustment could result in even thicker deposition, reduced resistance, and increased production speed. Moreover, studying the effect of dip coating cycles on coating thickness and conductivity will be of interest to applications where a larger amount of MXenes will be needed, such as for energy storage applications.

In this study, we focused the proof-of-concept demonstration of these electrodes for electrical stimulation, electrical sensing, and H_2_O_2_ sensing for biointerfacing. However, these MXene microfiber electrodes can be used beyond these specific applications, wherever flexible, fiber-shaped electrodes and current collectors are required. For instance, we envision the possibility of using a thermally responsive polymeric filament as a substrate for photothermal actuated fibers, harnessing the exceptional photothermal conversion efficiency of MXenes. Furthermore, the functional groups on MXenes serve as effective anchoring points for binding antibodies and drugs, giving possibilities to transform the electrodes into vessels for controlled drug delivery. Due to their high electrical conductivity, these electrodes are promising for long-distance strain monitoring as smart sutures and smart composite reinforcements. While thicker MXene coatings are preferred for certain applications, thin coatings are preferred in other applications, such as gas sensing. Here, increased resistances and more exposed material to the gas result in greater resistance changes. The tunable and versatile nature of these MXene fiber electrodes unlocks a plethora of applications across various domains.

## Conclusions

In this study, we introduce a high-throughput approach for the swift fabrication of versatile microfiber electrodes using Ti_3_C_2_T_*x*_ MXene. The utilization of shear force in the high-speed coating process enables the adoption of viscous MXene dispersions at concentrations of up to 110 mg/mL. Through this method, MXene flakes are effectively aligned to produce coatings with a low resistance of ∼10 Ω/cm (electrical conductivity of 7093 ± 819 S/cm) after a single pass at minimal MXene consumption. This innovative process leads to the development of reliable, durable, and cost-effective Ti_3_C_2_T_*x*_ microfiber electrodes that are free of additives and current collectors. Furthermore, our adaptable methodology facilitates the easy customization of the electrode’s electrical, mechanical, and electrochemical properties by adjusting MXene concentration, filament diameter, and coating speed. These electrodes exhibit multifunctionality, enabling bidirectional electrical communication and H_2_O_2_ detection, as validated through *in vivo* and *ex vivo* studies. Additionally, the electrodes can be efficiently multiplexed for high-density and multimodal arrays and integrated with other investigative techniques such as MRI and optical stimulation. Applications in wearable electronics, where a fiber-shaped current collector or electrode is desired, can also be envisioned.

## Methods

### MXene Synthesis and Characterization

Ti_3_C_2_T_*x*_ was synthesized by etching of the Al layer from the MAX phase precursor Ti_3_AlC_2_ by using a mixture of hydrofluoric acid (HF) and hydrochloric acid (HCl), followed by delamination with an aqueous solution of lithium chloride (LiCl).^20^ Subsequent centrifugation yielded a stable aqueous dispersion of polydisperse, single-layer Ti_3_C_2_T_*x*_ flakes. The concentration of this dispersion was subsequently adjusted to a range of 10–110 mg/mL, achieved through either dilution or high-speed centrifugation. Dynamic Light Scattering (DLS) with a Nano ZS Zetasizer (Malvern Instruments) was employed to determine the flake size distributions and ζ-potential of the MXene dispersion. UV–vis spectra were also obtained using an Evolution 201 UV–vis spectrophotometer (Thermo Scientific) as proof of MXene synthesis quality. The viscosity of the dispersions was measured using a Discovery HR-3 rheometer (TA Instruments) at room temperature, employing a parallel plate configuration (plate diameter 40 mm). To ascertain the electrical conductivity of the synthesized MXene, the sheet resistance and thickness of a free-standing, vacuum-assisted filtration-obtained film were measured using a four-point probe setup (Jandel) and a micrometer, respectively. X-ray diffraction (XRD) analysis was performed on the same free-standing film as additional evidence of the successful synthesis of Ti_3_C_2_T_*x*_. The analysis was carried out using a SmartLab (Rigaku) with a Cu Kα (λ = 0.1542 nm) source and a graphite K_β_ filter, operated at a 40 kV voltage and 15 mA current.

### Fabrication of MXene Coated Filaments and Characterization

The nylon monofilaments ranging from 100 to 300 μm in diameter and multifilament nylon yarns were purchased from The Thread Exchange. To further reduce the electrode diameter, we isolated single filaments (28 μm in diameter) from a multifilament nylon yarn. The filament coating process began by threading the loose end of a nylon filament from the spool through a needle tip (gauge 14-15G) and then inserting it into a 2 mL round-bottom polypropylene graduated microcentrifuge tube filled with MXene solution. This tube was securely positioned in the lower grips of a 3382A universal tension machine (Instron) with a load cell of 50N, while the loose end of the nylon filament was clamped by the upper grips. As the upper grips moved at a predetermined speed, a thin layer of MXene coating was deposited onto the filament. The upper grips were allowed to reach their maximum height on the 1 m frame and then stopped to permit the coating to air-dry for 5 min. The morphology of the MXene coating was analyzed using a VK-X1000 optical profilometer (Keyence) and a Apreo 2S Lo Vac scanning electron microscope (Thermal Fisher). For SEM imaging, the samples were sputtered with platinum/palladium at 30 mA for 30 s using a sputter coater 108 auto (Cressington Scientific) to prevent sample charging. Cross sections of the MXene-coated filaments were exposed for SEM imaging by cutting them with a fresh blade against a glass substrate at room temperature. To measure the electrical resistance of the MXene-coated filaments, a two-point probe method was utilized with a hand-held multimeter (Klein Tools). Two small stainless steel flat-mouth alligator clips (5.59 mm mouth opening) were soldered, using 60:40 Tin–Lead (Sn/Pb) solder wire, to the probes on the two test leads that were plugged into the multimeter. The alligator clips secure a nonslip, secure contact between the probe and conductive fiber while allowing for easy accommodation of fibers of different diameters. With the clips positioned 1 cm apart along the coated filament, 10 resistance measurements were taken for each sample at different locations and then averaged. The maximum MXene loading (mg/cm) achievable with a single MXene dip coating was determined by weighing 100 cm of MXene-coated filaments (300 μm, 110 mg/mL, 15 mm/s). The weight of 100 cm of pristine 300 μm filament was subtracted from this, and the result was then divided by 100 cm. Note that electrical resistance was selected as the property to track in the parametric study because it can be measured more quickly and accurately than weight or thickness. MXene loading or weight-based measurements were not inaccurate for thinner coatings of less than 1 μm, even at a long sample length of 100 cm. The electrical conductivity of the MXene coating was determined by approximating the coating as a free-standing film with a length (*L*) of 1 cm, width (*W*) equivalent to the perimeter of the nylon filament, and thickness (*t*) representing the coating thickness. Using this method, length/width ratios (*W*/*L*) of 10.6, 15.9, and 31.8 were derived for filaments with diameters of 300, 200, and 100 μm, respectively. The resistivity (ρ) of the coating was calculated by , from which the electrical conductivity (σ) was obtained as . Rocking curve XRD was performed with the same Miniflex II–Gen. Six (Rigaku) to probe the alignment state of flakes in the coating.

### Encapsulated Electrodes Preparation

To make the electrodes, MXene-coated nylon filaments were cut into 5 cm segments and connected to stainless steel alligator clips using silver paste. These electrodes were aligned in parallel and coated with a 10 μm layer of Parylene C using a PDS2010 Parylene coater (Specialty Coating Systems). The MXene coating was exposed for testing by carefully cutting the electrode tip with a fresh blade against a glass substrate. The Parylene C encapsulation thickness was determined to be 10 μm, as thinner coatings do not provide adequate mechanical strength for cutting at room temperature. A 10 μm Parylene C coating provides a consistent amount of exposed MXene in the cross section after cutting, while thinner Parylene C coatings of 2 and 5 μm occasionally tear, leading to nonuniformities of the exposed MXene.

### Bending Stiffness Estimation of the Electrodes

The bending stiffness (*K*) of the electrodes was computed using a core-double shell cylindrical model with the following equation^80^
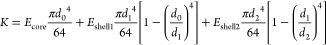
where *E*_core_ is the Young’s modulus of nylon, *d*_0_ represents the diameter of the nylon filament, *E*_shell1_ is the Young’s modulus of the MXene coating, *d*_1_ represents the total diameter after MXene coating, *E*_shell2_ is the Young’s modulus of Parylene C, *d*_2_ represents the total diameter after Parylene C encapsulation. We used 2.25, 20.6, and 3.17 GPa for the Young’s modulus of nylon,^81^ MXene coating,^58^ and Parylene C,^82^ respectively. The results demonstrated that the bending stiffness of the electrodes is primarily dictated by the nylon substrate, with stiffness decreasing as the diameter decreases.

### Electrochemical Characterization of the Electrodes

A three-electrode setup was utilized, comprising a working electrode (MXene-coated), a reference electrode as Ag/AgCl (3 M KCl), and a platinum wire as the counter electrode. Two electrolytes were prepared: 5 mM ruthenium hexamine (Sigma-Aldrich) in 1 M KCl and 1× PBS (tablets, Sigma-Aldrich). 1× PBS was prepared by dissolving a tablet in 200 mL of deionized water. This setup was employed for conducting CV and EIS characterizations of MXene electrodes with varied parameters in both electrolytes. The characterizations were carried out using a VMP3 electrochemical workstation (BioLogic). To assess the reproducibility of the electrodes, cyclic voltammograms were recorded for five different MXene-coated fibers, of both coating concentrations of 10 and 110 mg/mL. Furthermore, to confirm the uniformity of MXene coating and electrode performance throughout the fibers, five different cross-sectional cuts were made along the fiber using a surgical blade, and recordings were made for each cross-sectional cut. For knotability assessment, the MXene electrode was manually knotted using tweezers and stretched to ensure a tight knot. For durability during insertion, a material with mechanical properties similar to those of a mammal brain was required. For this purpose, agarose gel was prepared by mixing agarose (Sigma-Aldrich Corporation) with deionized water at a concentration of 0.6% w/w, adding a drop of red food coloring for imaging, microwaving the mixture until boiling, and leaving it at room temperature to gel for at least 2 h. An electrode was inserted repeatedly into the agarose gel 3–4 cm deep, with CV and EIS data collected every 200 insertions.

### Assessment of H_2_O_2_ Sensing Capabilities

Amperometry experiments were conducted for both MXene electrodes and carbon fiber at −0.65 V vs Ag/AgCl with in situ additions of H_2_O_2_ in a 1× PBS solution. The additions resulted in H_2_O_2_ concentrations of 20, 50, 100, 200, and 500 μM. These concentrations were then plotted against the current to determine the sensitivity of the electrodes, which was calculated from the slope of the line. To assess measurement stability, the applied potential was held for 30 min after the addition of 1 mM H_2_O_2_. Additionally, measurements of 1 mM H_2_O_2_ were tested amperometrically over 4 consecutive days to further evaluate the stability of electrode performance.

### *Ex Vivo* H_2_O_2_ Sensing in Murine Bladder Urothelium

All procedures were performed according to the regulations of the United Kingdom Home Office and the Animals (Scientific Procedures) Act, and they were approved by the Animal Welfare and Ethical Review Body at the University of Brighton. Wildtype C57BL/6J male mice were euthanized using CO_2_ gas followed by cervical dislocation. The entire bladder was then removed and placed in H medium, with a pH of 7.4 (composition: 145 mM NaCl, 5 mM KCl, 1 mM MgCl_2_, 0.8 mM CaCl_2_, 10 mM Hepes, and 5 mM glucose). The bladder was cut open longitudinally, pinned into a Sylgard lined flow bath, and constantly preferred with H medium. The MXene fiber electrode was placed on the surface of the bladder and held at −0.65 V vs Ag/AgCl. Hydrogen peroxide production was stimulated using 200 nM thapsigargin through microperfusion.

### Voltage Transient Measurements of the Electrodes

Charge injection characterization of the MXene microfiber electrodes was performed via voltage transient measurements in a 3-electrode electrochemical cell using a Gamry R600 potentiostat. MXene microfiber electrodes, an Ag/AgCl electrode, and a carbon electrode were used as the working, reference, and counter electrodes, respectively. Asymmetric biphasic current pulses with incrementally increasing current magnitudes were applied at the working electrode. The cathodic, interpulse, and anodic pulse durations were fixed at 500, 100, and 1000 μs. The cathodic potential excursion was defined as the electrode potential vs Ag/AgCl after the end of the cathodic phase of the current pulse (*I*_cat_ → 0 A). The magnitude of the charge injected was calculated as the time integral of the injected charge per cathodic pulse. The charge injection capacity (CIC) was calculated as the area-normalized charge injected at a cathodic excursion potential of −1.3 V vs Ag/AgCl for MXene microfibers.

### *In Vivo* Electrical Recording and Stimulation Using MXene Microfiber Electrodes

All procedures were approved by the Institutional Animal Care and Use Committee at the University of Pennsylvania. Male Sprague–Dawley rats (Charles River Laboratories; 300–330 g; aged 6–8 weeks) were anesthetized with isoflurane (5% induction, 1.5–2% maintenance); the hind leg was shaved and cleaned with betadine solution. Meloxicam (2 mg/kg) and bupivacaine (2 mg/kg) were administered subcutaneously in the scruff of the neck and along the incision, respectively. The sciatic nerve was exposed by separating the gluteal muscle. The exposed sciatic nerve was stimulated via symmetric biphasic current pulses of varying amplitudes (0–200 μA), pulse duration of 100 μs, and frequency of 1 Hz (A-M Systems Isolated Pulse Stimulator Model 2100). Electrical stimulation was applied in a bipolar configuration (commercially available bipolar W electrodes, Rochester Electro-Medical, Lutz, FL; #400900, and paired MXene microfiber electrodes). The evoked electromyography (EMG) activity was recorded subdermally over the tibialis anterior using a bipolar configuration with the primary recording electrode placed over the muscle, the reference electrode placed in the tendon, and the ground electrode placed subcutaneously (50 kHz sampling rate; 100× gain and 1–10 000 Hz band-pass; A-M Systems Microelectrode AC Amplifier Model 1800). For recruitment curves, evoked EMG recordings were obtained by gradually increasing the stimulation current amplitude until the amplitude of the evoked EMG plateaued. For each current amplitude, a train of 10 individual stimulation pulses was applied.

### Surface Electromyography Data Acquisition and Analysis

Before placing a MXene coated nylon fiber on a healthy volunteer, the skin over the biceps muscle belly was cleaned with an alcohol wipe and moisturized with a few drops of 1× PBS. A 300 μm diameter MXene-coated fiber electrode (80 mg/mL MXene coated at 15 mm/s) was then placed at the target location with an Ag/AgCl reference and ground electrodes (Natus) placed on the bony part of the elbow and the wrist, respectively. sEMG activity was recorded in a monopolar configuration at 5 kHz using a commercial amplifier (Intan RMS2000, Intan Technologies).

Raw sEMG data was bandpass filtered (20–450 Hz) with a fourth order Butterworth filter, and noise due to 60 Hz and its harmonics was filtered out using iterative notch filters. The root-mean-square (RMS) of the filtered sEMG data was calculated over 200 ms epochs with a 50 ms overlap.

### Statistical Analysis

All values with error bars represented means ± standard deviations (*n* ≥ 5) and were analyzed using Excel software and plotted using Originlab software. Main factor and interaction plots were conducted and generated using Minitab software. Electrophysiology data analysis was performed using Matlab. Briefly, digital notch filters were applied to remove noise due to 60 Hz and harmonics. For individual stimulation pulses, the recorded evoked EMG was segmented and averaged. Peak-to-peak EMG was calculated as the difference between the maximum and minimum amplitudes of the evoked EMG.

## Data Availability

The data supporting the findings of this study are available upon reasonable request from the authors.

## Supporting Information Available

Coating setup and examples of meniscus formations by withdrawing nylon fibers from MXene solutions of different concentrations (Movies S1) (MP4)
Ti_3_C_2_T*_x_* characterizations; rheology of MXene suspensions in water; standard deviation and main factor analysis of resistances; SEM images, EDS maps, and optical images of nylon filaments (without parylene C encapsulation); liquid crystalline calculations; rocking curve XRD setup and analysis; figure of merit of linear resistance and bending stiffness of MXene-coated nylon filaments, compared to fiber electrodes of other materials in the literature; images demonstrating assistance-free insertion; SEM images and EDS maps of nylon filaments (with parylene C encapsulation); schematics of 3-electrode setup for electrochemical characterization; CV and EIS spectra measured in RuHex of MXene fiber electrodes of different specifications; images and figures showcasing the knotability, durability, long cyclability, and shelf life of MXene fiber electrodes; CV of a carbon fiber electrode for comparison; voltage transients and cathodic charge injection capacity CICc of MXene fiber electrodes; photos of the In vivo experiment on a rat with MXene fiber electrodes and the recorded electromyography responses; images demonstrating the versatility of the reported coating method; tables of linear resistances and conductivities of MXene fiber electrodes and silver-plated nylon filaments; tables of bending stiffness, impedance, CICc, and CSCc of MXene-nylon-Parylene C fiber electrodes compared to fiber electrodes made of other materials in the literature (PDF)
