# Supplementary material for: Soft, Multifunctional MXene-Coated Fiber Microelectrodes for Biointerfacing
Source: ACS Nano. 2024 Aug 14;18(34):23217–31. doi: 10.1021/acsnano.4c05797 (PMC11363215; doi:10.1021/acsnano.4c05797)
Supplement: Supplementary file 2 — nn4c05797_si_002.pdf [file nn4c05797_si_002.pdf]

Supporting Information

Soft, Multifunctional MXene-Coated Fiber Microelectrodes for Biointerfacing

*Lingyi Bi[1], Raghav Garg[2], Natalia Noriega[3], Ruocun (John) Wang[1], Hyunho Kim[1], Kseniia Vorotilo[1], Justin C. Burrell[6], Christopher E. Shuck[1], Flavia Vitale[2,4,5], Bhavik Anil Patel[3], Yury Gogotsi[1]**

[1] Department of Materials Science and Engineering and A. J. Drexel Nanomaterials Institute, Drexel University, Philadelphia, PA, 19104, USA

[2] Department of Neurology, University of Pennsylvania, Philadelphia, PA, 19104, USA

[3] School of Applied Sciences, University of Brighton, Brighton, BN2 4AT, UK

[4] Department of Bioengineering, University of Pennsylvania, Philadelphia, PA, 19104, USA

[5] Department of Physical Medicine and Rehabilitation, University of Pennsylvania, Philadelphia, PA, 19104, USA

[6] Department of Oral and Maxillofacial Surgery & Pharmacology, University of Pennsylvania School of Dental Medicine, Philadelphia, PA, 19104, USA

* E-mail: gogotsi@drexel.edu

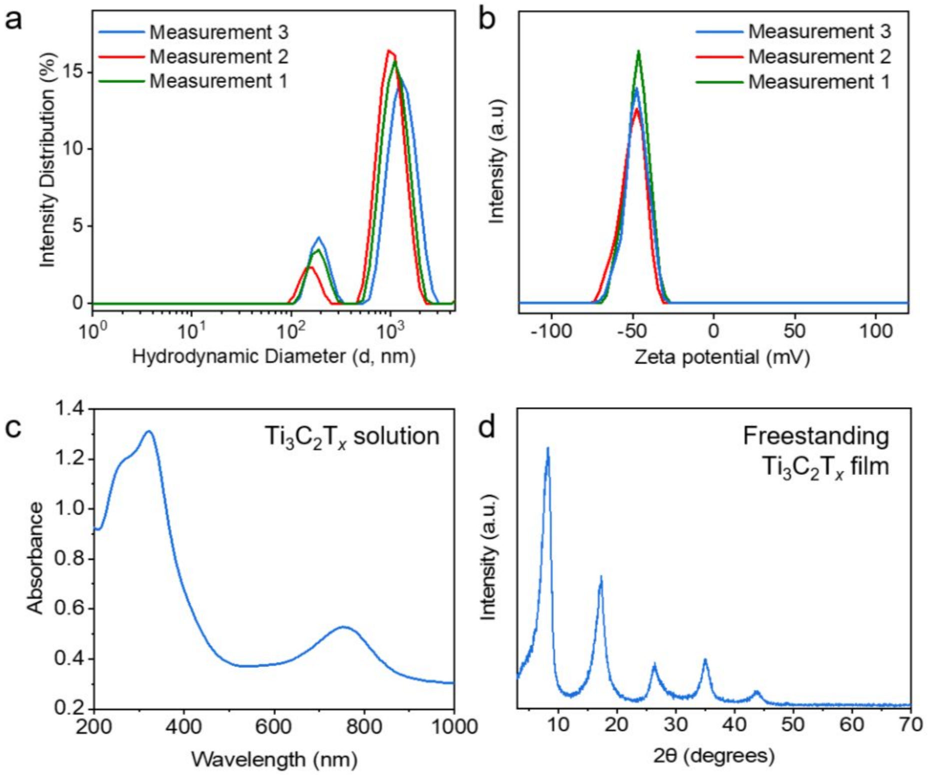

Figure S1. $Ti_3C_2T_x$ characterization. Measurements of a) flake size (n=3) and b) zeta potential (n=3) via dynamic light scattering (DLS). c) UV-vis of diluted $Ti_3C_2T_x$ solution (concentration < 0.01 mg/mL) and d) XRD pattern of freestanding $Ti_3C_2T_x$ film obtained from vacuum-assisted filtration, both confirming the successful synthesis of $Ti_3C_2T_x$.

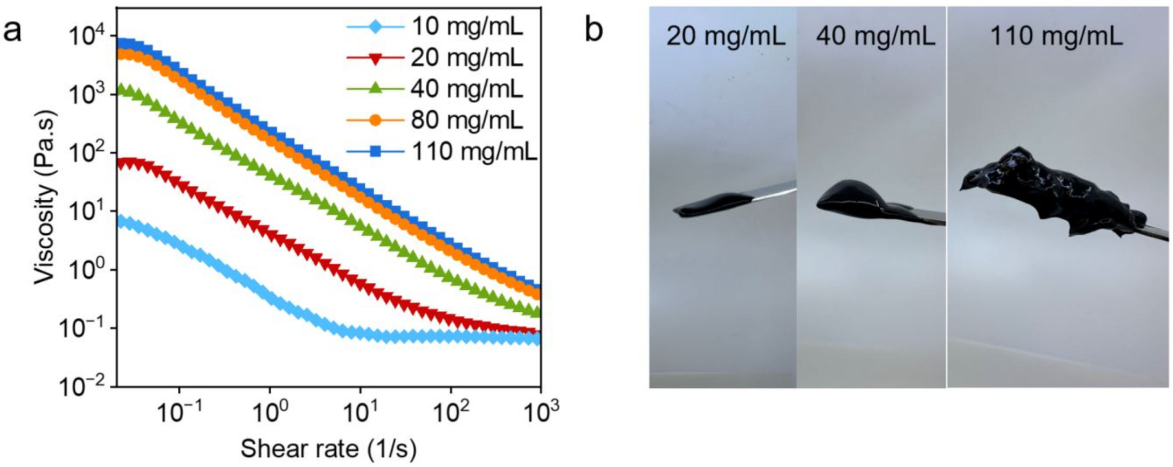

Figure S2. Rheology of MXene suspensions in water. a) Relationship between viscosity and shear rate for $Ti_3C_2T_x$ colloidal dispersions at concentrations ranging from 10 to 110 mg/mL. b) Digital images showing the consistency of $Ti_3C_2T_x$ dispersions at selected concentrations when scooped with a spatula.

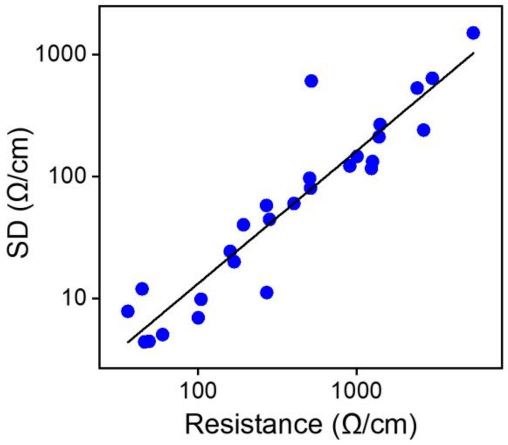

Figure S3. Standard deviation versus average linear resistance of MXene-coated nylon filaments with their least squares regression line.

Main factor analysis

Averaging resistances per parameter revealed that MXene concentration and filament diameter exert a more substantial influence than drawing speed, as evidenced by their steeper slopes and wider range coverage of resistances, following the factor analysis methodology (Figure S4, Supporting Information). To further illustrate parameter interactions, we depicted the mean resistance of one parameter on the x-axis, with a separate line for each level of a second parameter (Figure S5, supporting information). The multiple crossings or potential crossing points between the lines indicate intricate interactions among the three parameters. The interactions can be attributed to the complexity of the fluidics in the meniscus and the rheology of MXene.

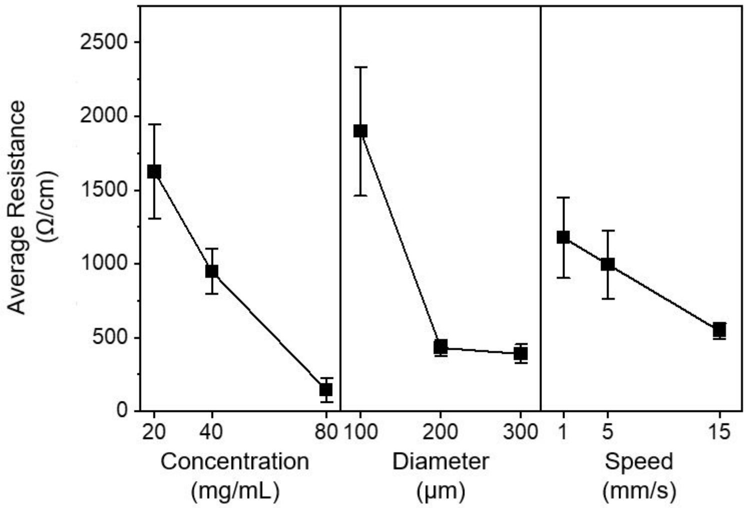

Figure S4. Main effects plots presenting the mean resistance for each parameter, thus combining the effects of the other two parameters, offering insight into the relative impact of each parameter on resistance.

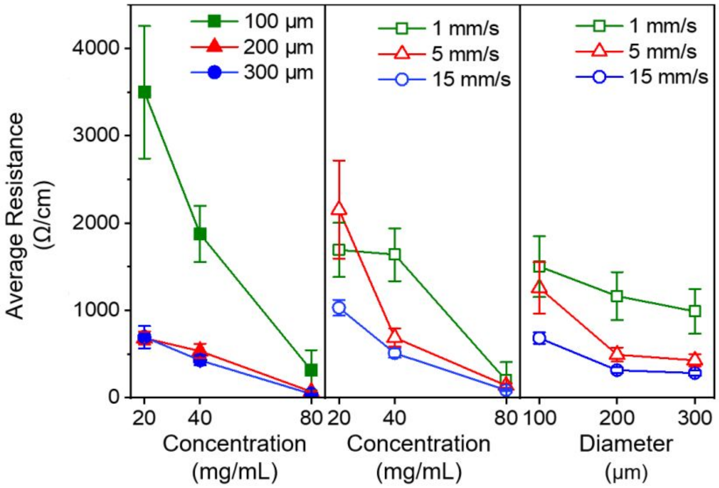

Figure S5. Parameter interaction plots, with the mean resistances for the levels of one parameter presented on the x-axis and a separate line for each level of a second parameter.

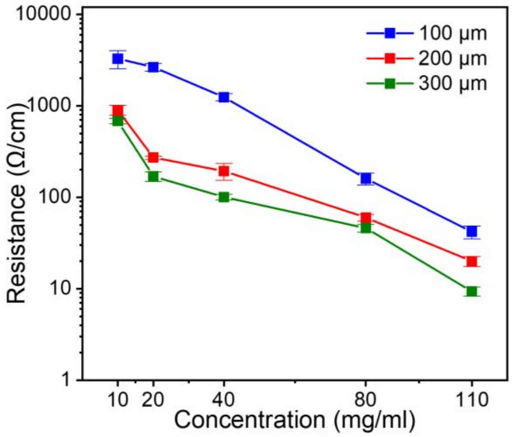

Figure S6. The change in linear resistance and SD with $Ti_3C_2T_x$ concentrations for 10 mg/mL to 110 mg/mL for electrodes of different diameters from 100 to 300 µm.

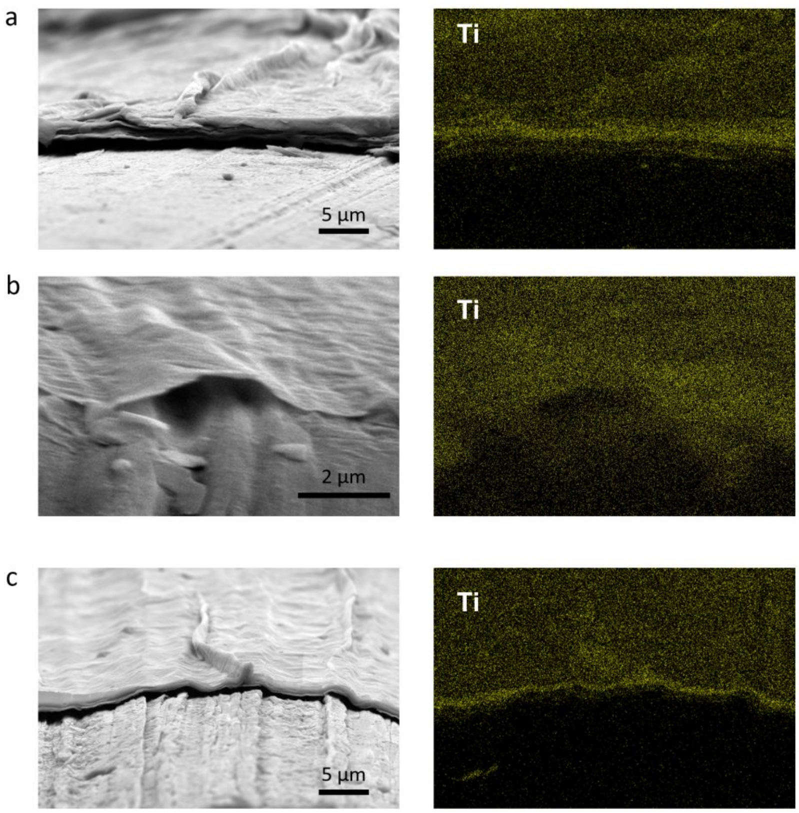

Figure S7. SEM images and EDS maps of nylon filaments of a) 300 µm diameter coated with 110 mg/ml MXene solution, b) 300 µm diameter coated with 10 mg/ml solution, c) 100 µm diameter coated with 110 mg/mL solution. All samples were coated at the speed of 15 mm/s. The EDS images were net counts with brightness increased by 60 %, 70 %, and 60 %, respectively, to improve clarity.

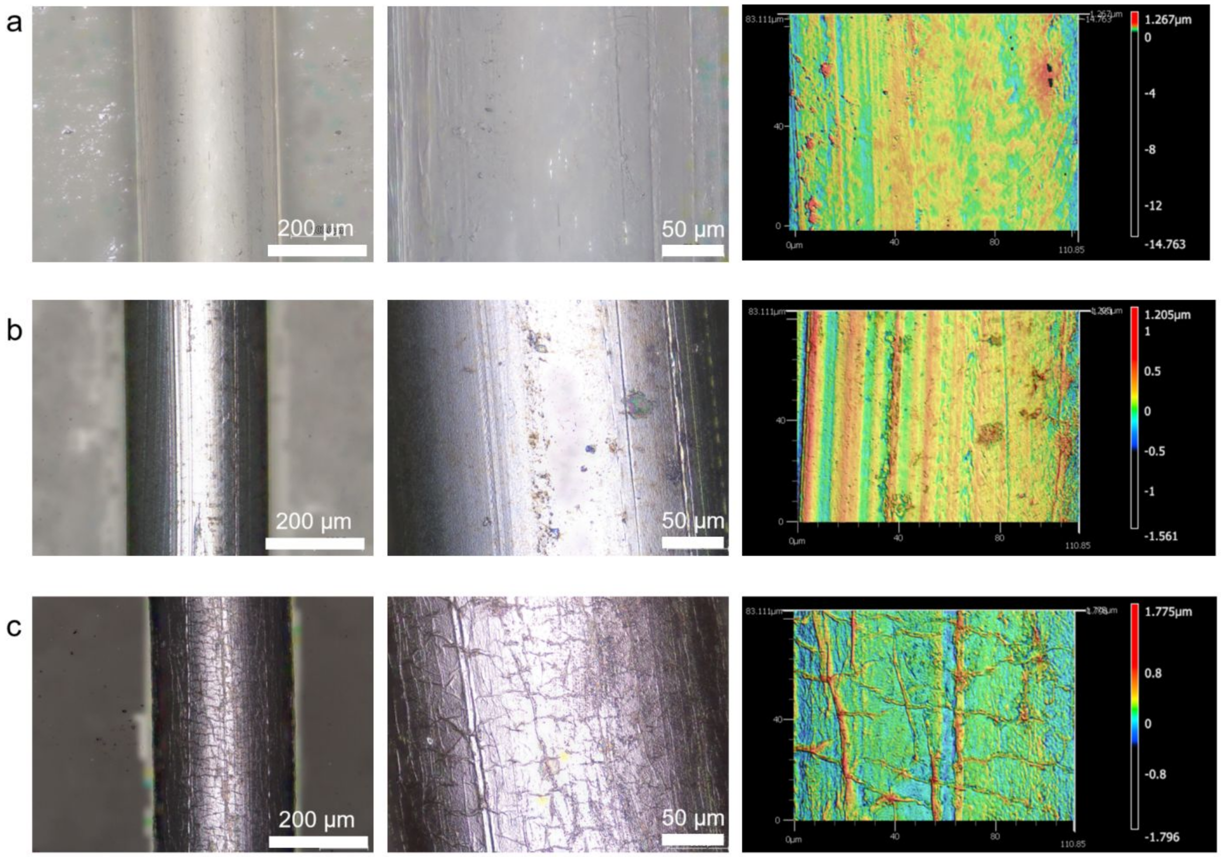

Figure S8. Optical images of a) 300 µm pristine nylon, b) 300 µm, 10 mg/ml, 15 mm/s – ultra-thin coating, with the nylon features still showing through, c) 300 µm, 110 mg/ml, 15 mm/s – thicker coating, with wrinkles formed from shrinkage during drying.

Liquid-Crystalline MXene and Rocking Curve XRD Analysis

The calculations for liquid crystalline concentration were carried out following prior research, where the theoretical MXene concentration (c) at the isotropic-nematic transition, calculated by $c = 1.03\frac{\pi\rho}{\alpha}$, demonstrated good agreement with experimental data.[6] In the calculation, a $Ti_3C_2T_x$ density (ρ) of 5.15 g/cm^3 was adopted. The aspect ratio (α) was obtained by dividing the MXene width (W) by its thickness (t), where W is the intensity-weighted mean hydrodynamic size (Z average) of flakes from DLS and t is 1 nm. Following the example, based on the average flake size of the $Ti_3C_2T_x$ synthesized for this study, a theoretical MXene concentration of 16.6 mg/mL at the isotropic-nematic transition was calculated. This suggests the 110 mg/mL MXene solution has liquid crystalline ordering while the 10 mg/mL solution does not. We hypothesized that the spontaneous formation of a nematic liquid crystalline state in the 110 mg/mL solution would lead to coatings with superior flake alignment, akin to the results previously observed in wet spinning and blade coating processes.[4,6]

To confirm the hypothesis, we employed rocking curve XRD data to investigate the flake alignment (Figure S9, supporting information). The (002) peak of the 110 mg/mL MXene coated filament was more pronounced than that of the 10 mg/mL, indicating a coating with a higher degree of alignment is achieved with the elevated liquid crystalline forming concentration (Figure S10, supporting information). Such alignment, coupled with a thicker MXene deposit resulting from increased viscosity, contributes to a reduction in electrical resistance.

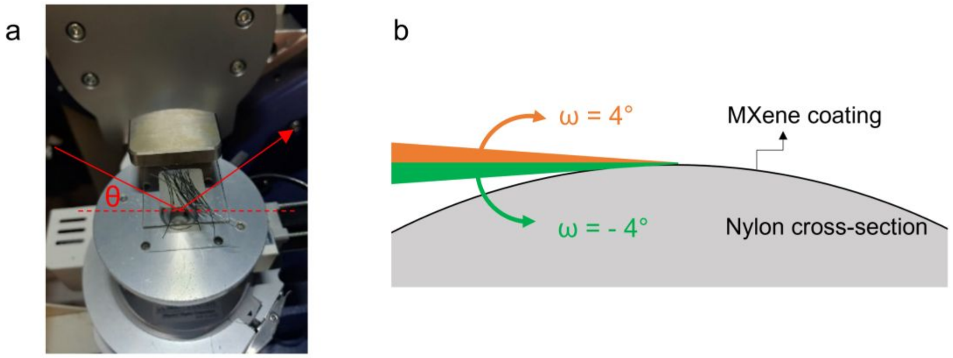

Figure S9. a) MXene fiber placement and b) range of stage rotation (- 4° to 4°) during rocking chair XRD measurement.

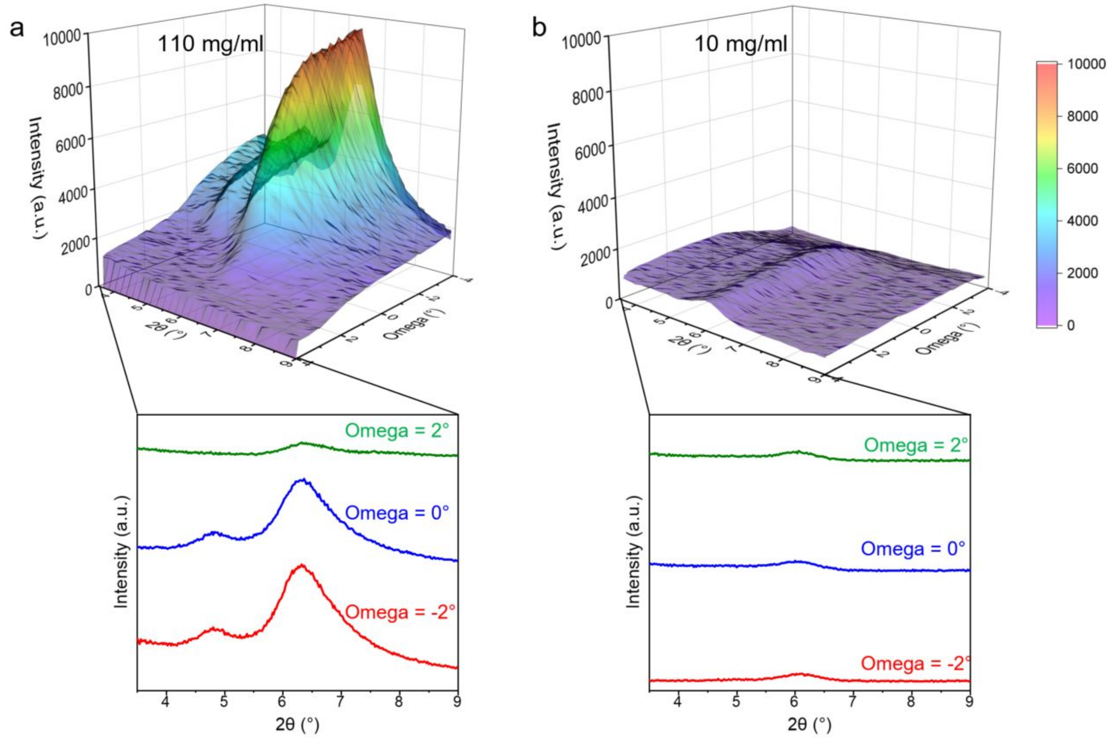

Figure S10. Rocking chair XRD of 300 µm nylon filaments coated with a) 110 mg/ml and b) 10 mg/ml MXene solution. The two (002) peaks of 110 mg/ml and 10 mg/ml are nearly at the same position. An additional layer of water was observed in 110 mg/ml MXene-coated filaments. This

is likely due to insufficient drying as the dried surface layer acts as a barrier for the moisture underneath.

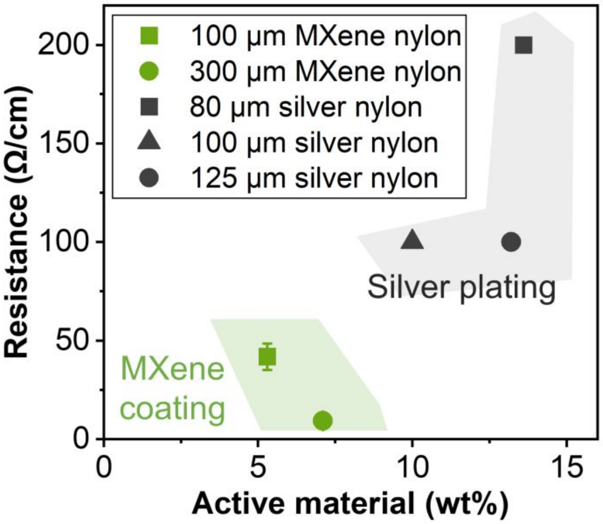

Figure S11. Linear resistance of MXene-coated nylon filaments and silver-plated nylon filaments (Shieldex®) as a function of the weight percentage of active material. Details can be found in Table S3.

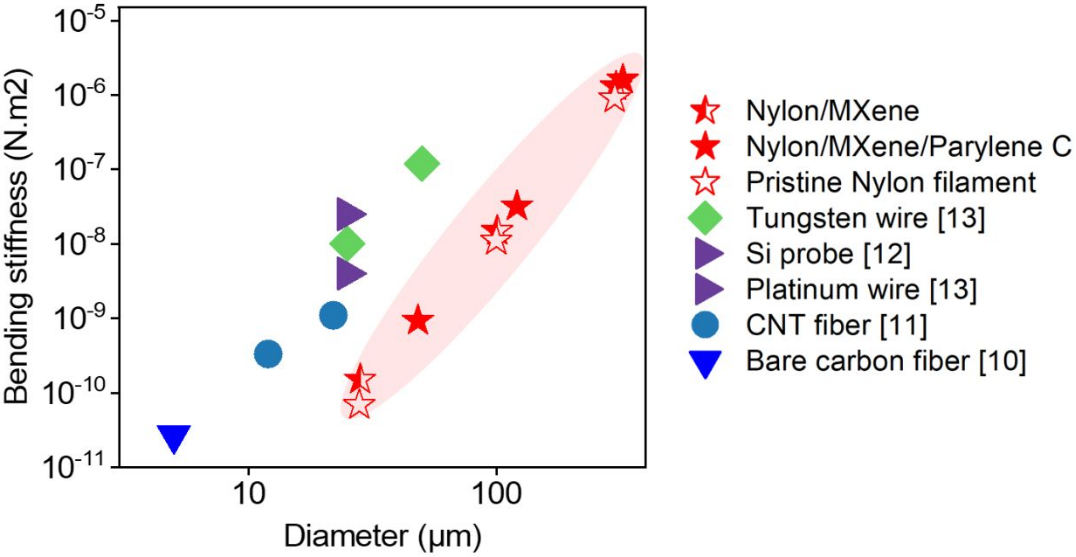

Figure S12. Bending stiffness of MXene-nylon-Parylene C fiber electrodes as a function of diameter compared to fiber electrodes of other materials in literature. Details can be found in Table S4.

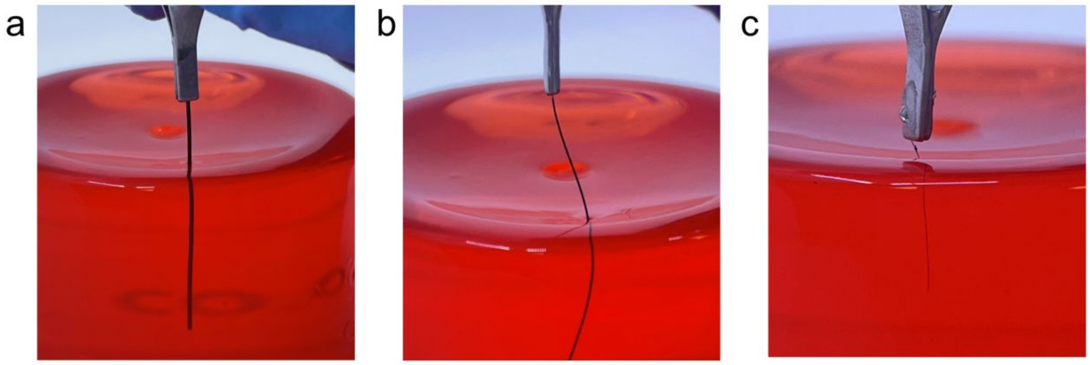

Figure S13. Assistance-free insertion of 110 mg/ml coated fiber electrodes of a) 300 µm, b) 100 µm, and c) 28 µm in 0.6 wt% agarose gel.

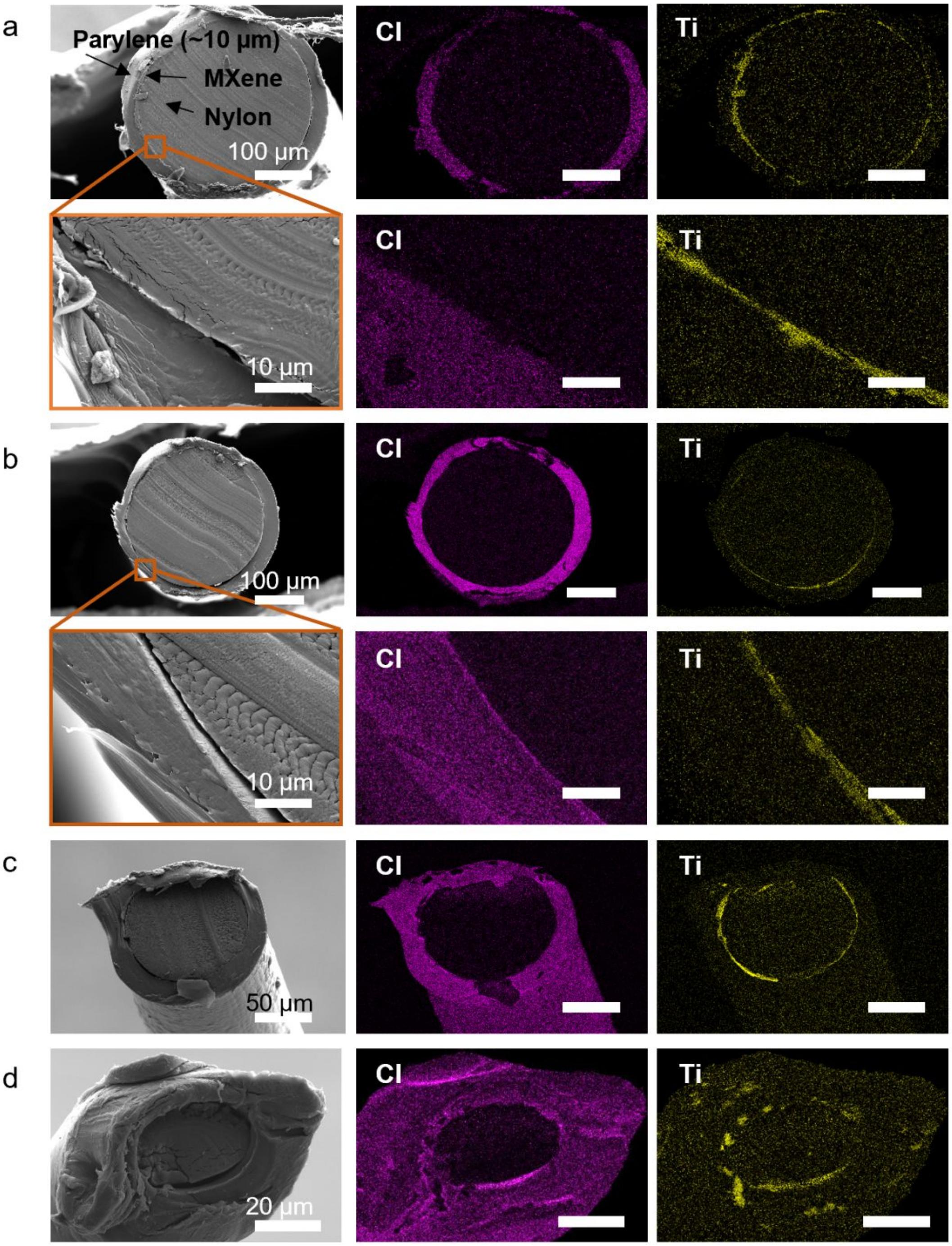

Figure S14. SEM and EDS of cross-sectional images of MXene-nylon-Parylene C fiber electrodes before deployment, after the ends were cut with a fresh blade against a glass substrate. a) 300 µm diameter electrodes coated with 110 mg/ml MXene solution, b) 300 µm diameter electrodes coated with 10 mg/ml MXene solution, c) 100 µm diameter electrodes coated with 110 mg/ml MXene solution, d) 28 µm diameter electrodes coated with 110 mg/ml MXene solution. All filaments were coated at the rate of 15 mm/s. The EDS images are based on the net count of the elements and increased with 50% brightness for clarity.

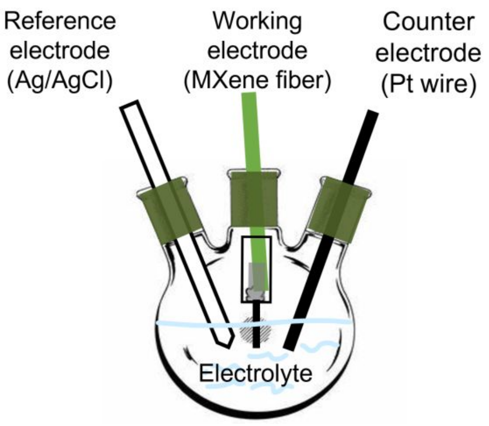

Figure S15. 3-electrode setup for MXene nylon electrode characterization.

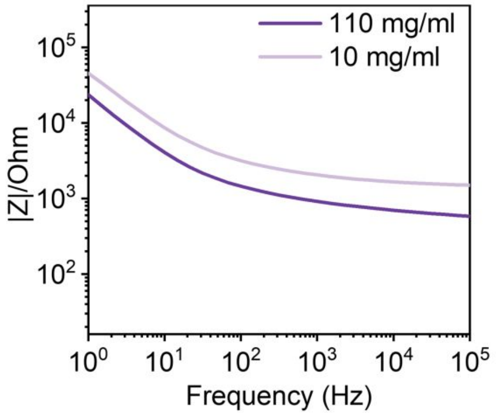

Figure S16. EIS spectra measured in RuHex using 300 µm diameter electrodes coated with 110 mg/ml and 10 mg/ml MXene dispersions.

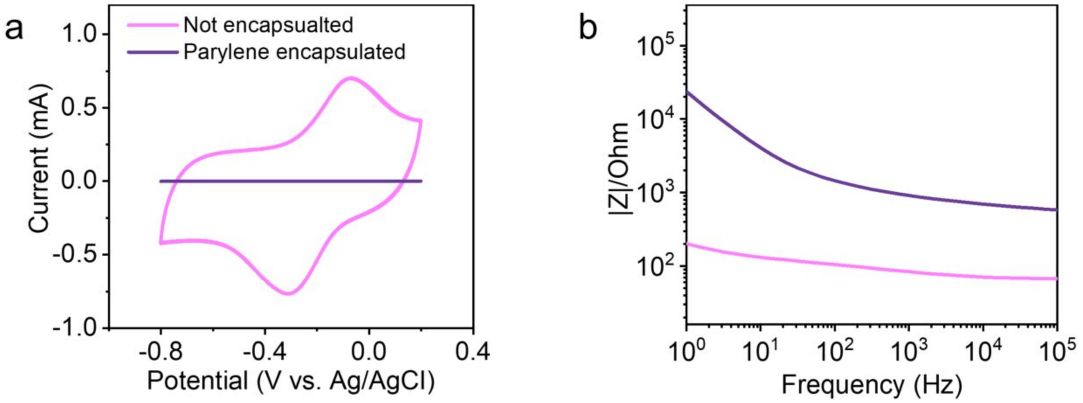

Figure S17. a) CV and b) EIS spectra of 300 µm diameter electrodes (110 mg/mL MXene, 15 mm/s) compared before and after Parylene C encapsulation in 5 mM RuHex in 1 M KCl at 20 mV/s scan rate.

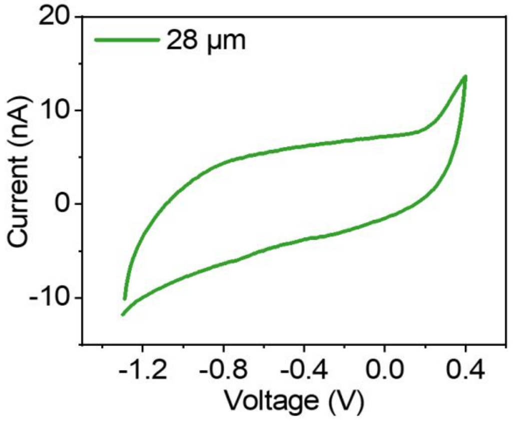

Figure S18. CV of a 28 µm electrode (110 mg/mL, 15 mm/s) in 1X PBS at 100 mV/s scan rate.

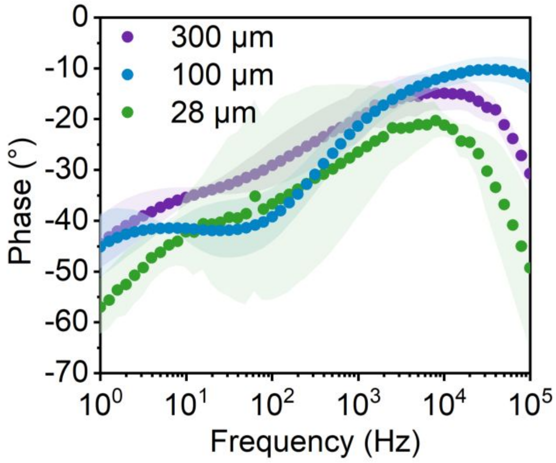

Figure S19. Phase angle of EIS of 100 mg/ml MXene coated electrodes of 28 µm, 100 µm and 300 µm diameter in 1X PBS. Data are plotted as means with shaded regions corresponding to SDs.

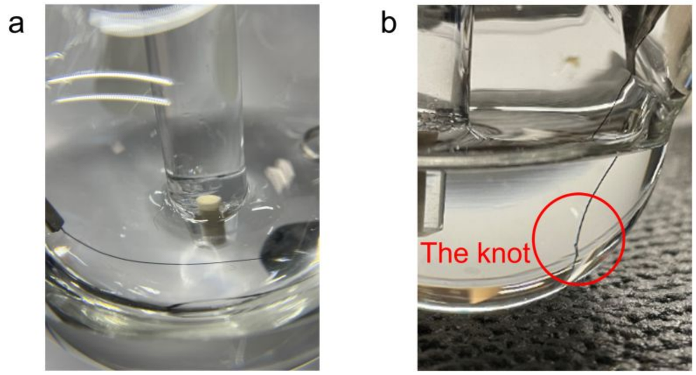

Figure S20. Digital photos of a) straight and b) knotted Nylon-MXene-Parylene C electrodes of 100 μm diameter, 110 mg/mL coated at 15 mm/s.

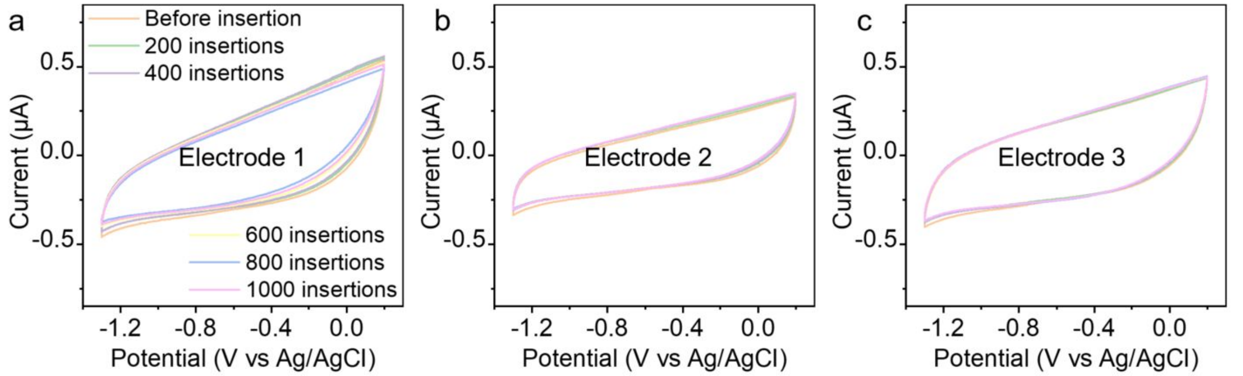

Figure S21. CV of electrodes are consistent after 1000 insertions into 0.6 wt% agarose (insertion depth 2 cm) in 1X PBS of 3 tested electrodes of 100 μm diameter.

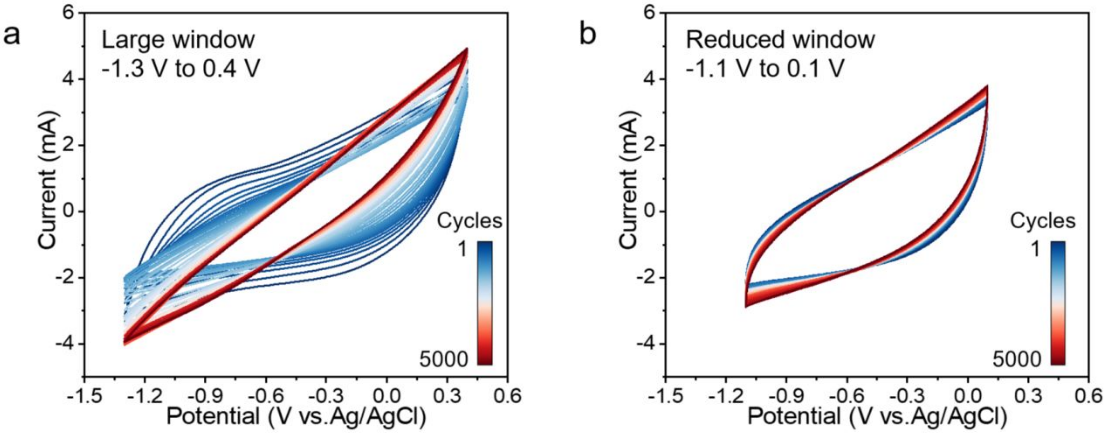

Figure S22. CV cyclability at 100 mV/s of 300 μm electrodes (110 mg/mL, 15 mm/s) under two windows: a) an extended voltage window of -1.3 V to 0.4 V and b) a narrowed voltage window of -1.1 V to -0.1 V, for 5000 cycles respectively.

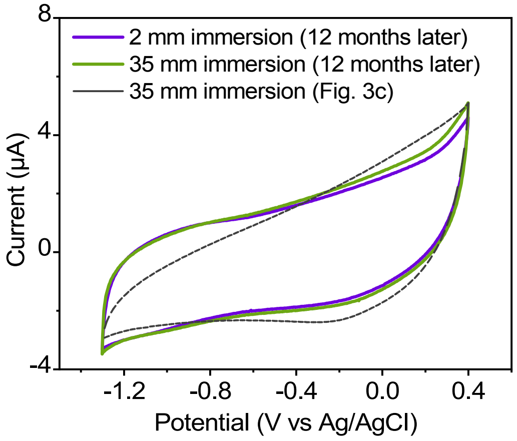

Figure S23. CVs of 300 µm electrodes (110 mg/mL, 15 mm/s) at three conditions of 1) tested right after fabrication at a 35 mm immersion depth, 2) after 12 months of bench storage at the same 35 mm immersion and 3) after 12 months of bench storage at a reduced 2 mm immersion. All 3 tests were conducted in 1X PBS at a scan rate of 100 mV/s.

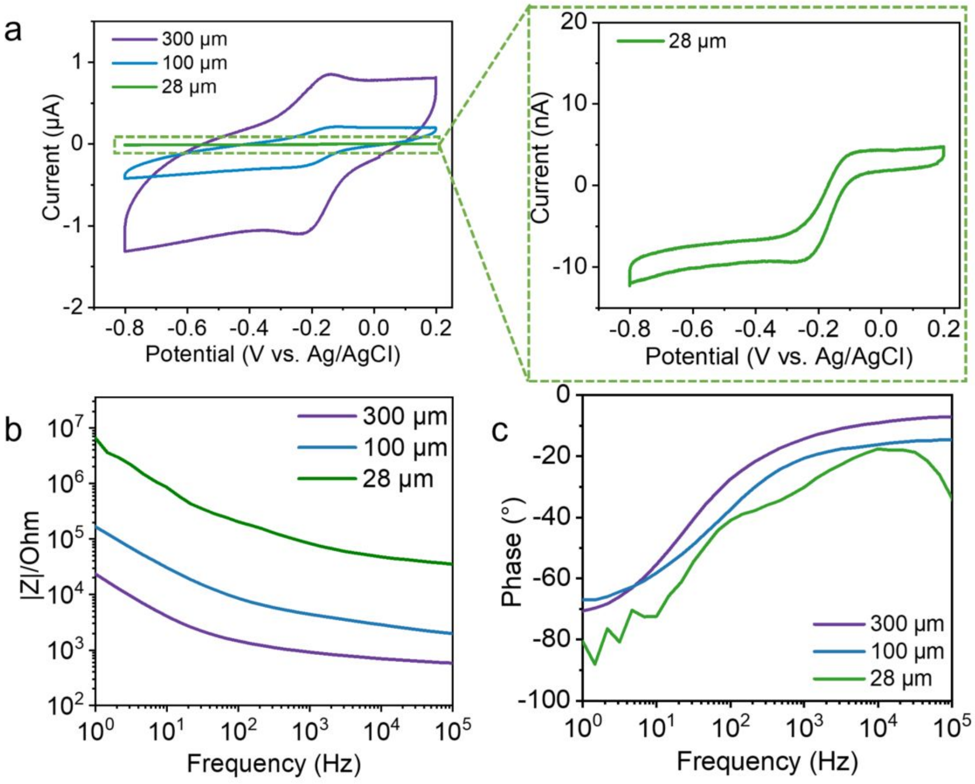

Figure S24. Electrochemical characterizations. a) CVs, b) impedance and c) phase angle of 110 mg/mL MXene coated electrodes of different diameters in 5 mM RuHex and 1 M KCl.

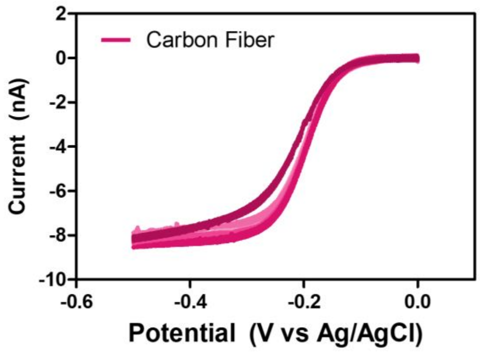

Figure S25. CV of a carbon fiber electrode in 5 mM RuHex and 1 M KCl at 20 mV/s.

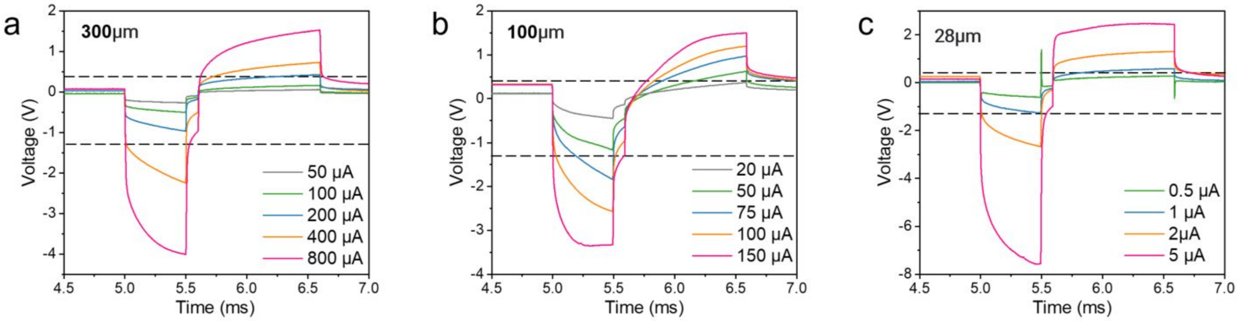

Figure S26. Voltage transients measured as a response to biphasic current pulses for 110 mg/ml MXene-coated electrodes of a) 300 µm, b) 100 µm, and c) 28 µm diameter. Dotted black lines denote the safe operating window of – 1.3 to 0.4 V.

Voltage transients recorded for 28 µm MXene coated nylon fiber and troubleshooting of abnormal spikes

For the most accurate measurement of the voltage transients, the sampling rate of the Gamry Reference 600 potentiostat was set to the fastest possible rate of 3.33 µs. As a result, for low values of applied current, the potentiostat tended to slightly overshoot, resulting in spikes at points of steep change in signal amplitude. We can observe such overshoots in the applied biphasic current pulse for the 28 µm diameter MXene-coated nylon fibers (Figure S27a, red curve). Thus, the emergence of such overshoots at the fastest acquisition settings is responsible for the sharp peaks observed in the voltage transient (Figure 27a, blue curve).

For higher current amplitudes, the potentiostat does not generate any overshoots when applying the specified current pulses (Figure S27b-d, red curves). Therefore, no steep spikes are observed in the corresponding voltage transients (Figure S27b-d, blue curves). Reducing the speed of data acquisition might prevent an overshoot in the applied current amplitude. However, we will lose the temporal resolution necessary for accurately determining the cathodic and anodic potential excursions.

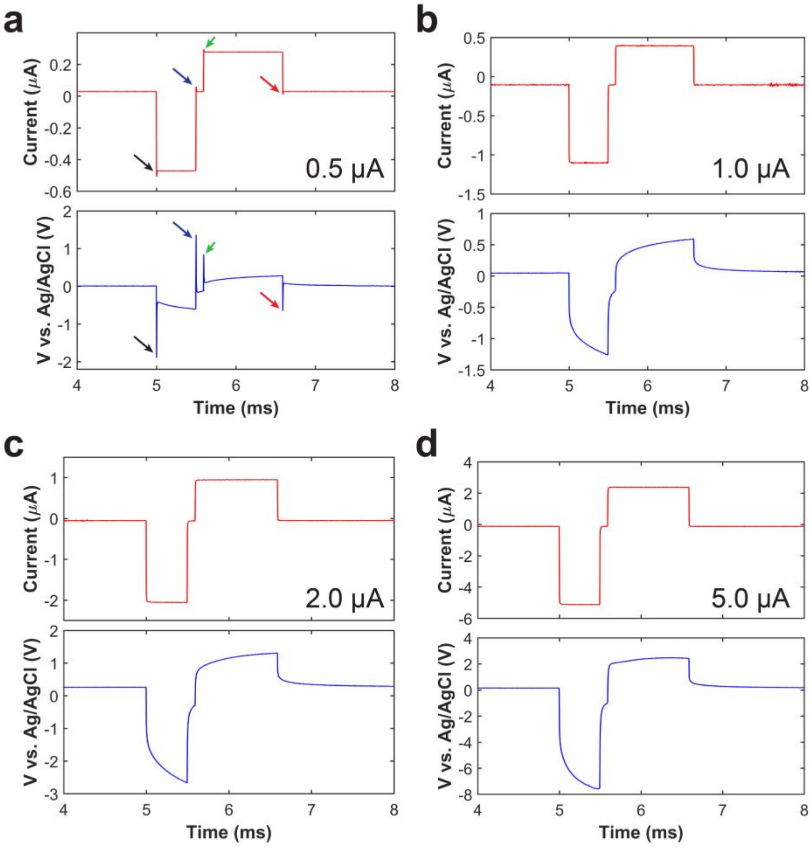

Figure S27. Voltage transients recorded for 28 µm MXene-coated nylon fiber and troubleshooting of abnormal spikes. Measured voltage transients (blue) and applied current pulses (red) for a representative 28 µm diameter $Ti_3C_2T_x$ MXene coated nylon fiber at current amplitudes of a) 0.5 µA, b) 1.0 µA, c) 2.0 µA, and d) 5.0 µA. Arrows in a) denote the spikes in the applied current pulse and the corresponding peaks measured in the voltage transient.

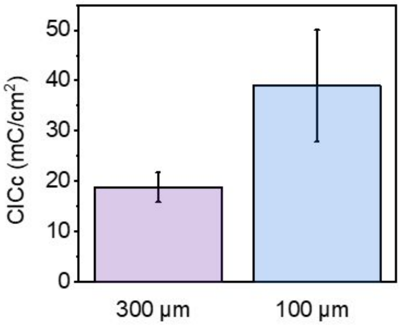

Figure S28. The cathodic charge injection capacity CICc when normalized by active material area for electrodes of diameters of 300 um and 100 µm.

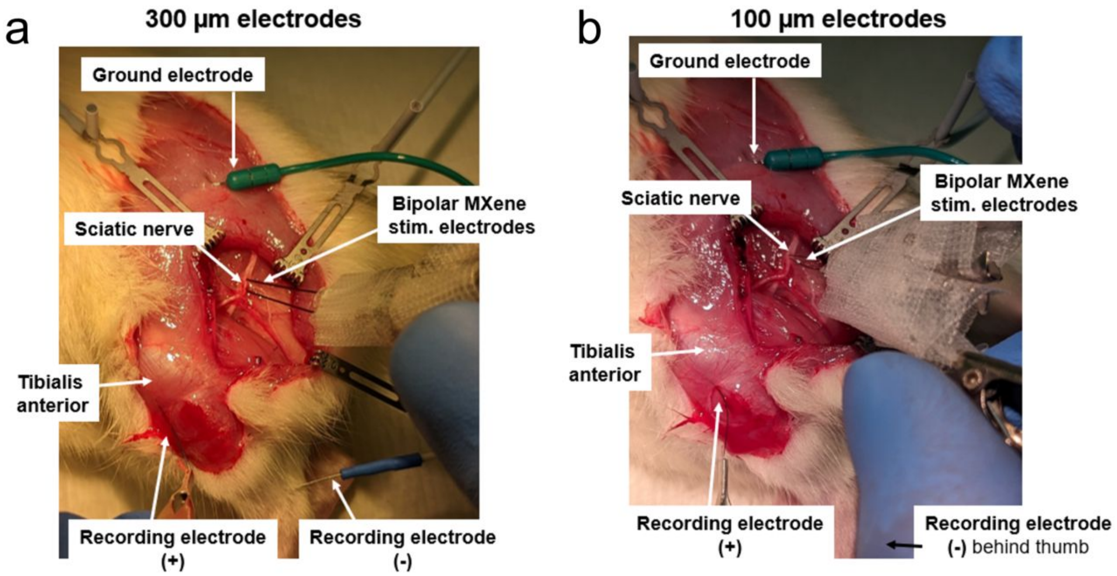

Figure S29. Photos of the *In vivo* experiment on a rat with a) 300 µm and b) 100 µm diameter electrodes.

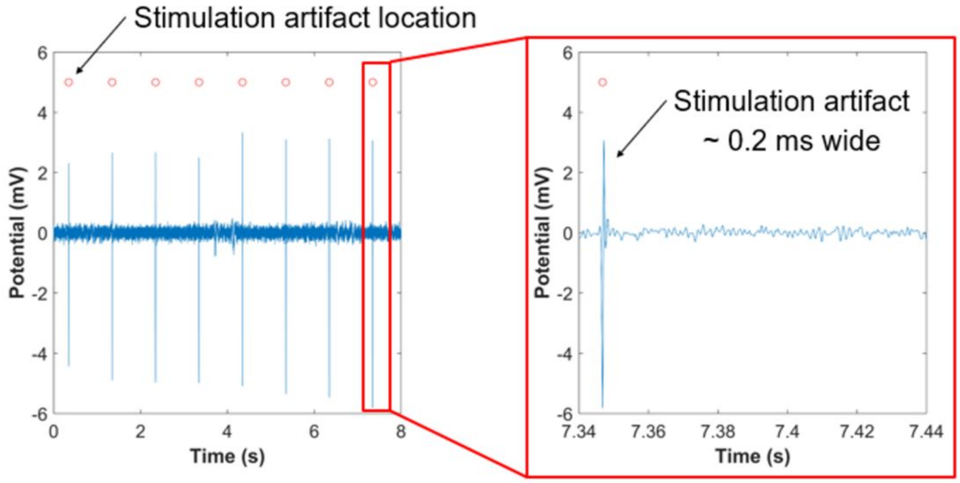

Figure S30. Identifying the location of the stimulation artifact as recorded by the electrophysiology recording setup.

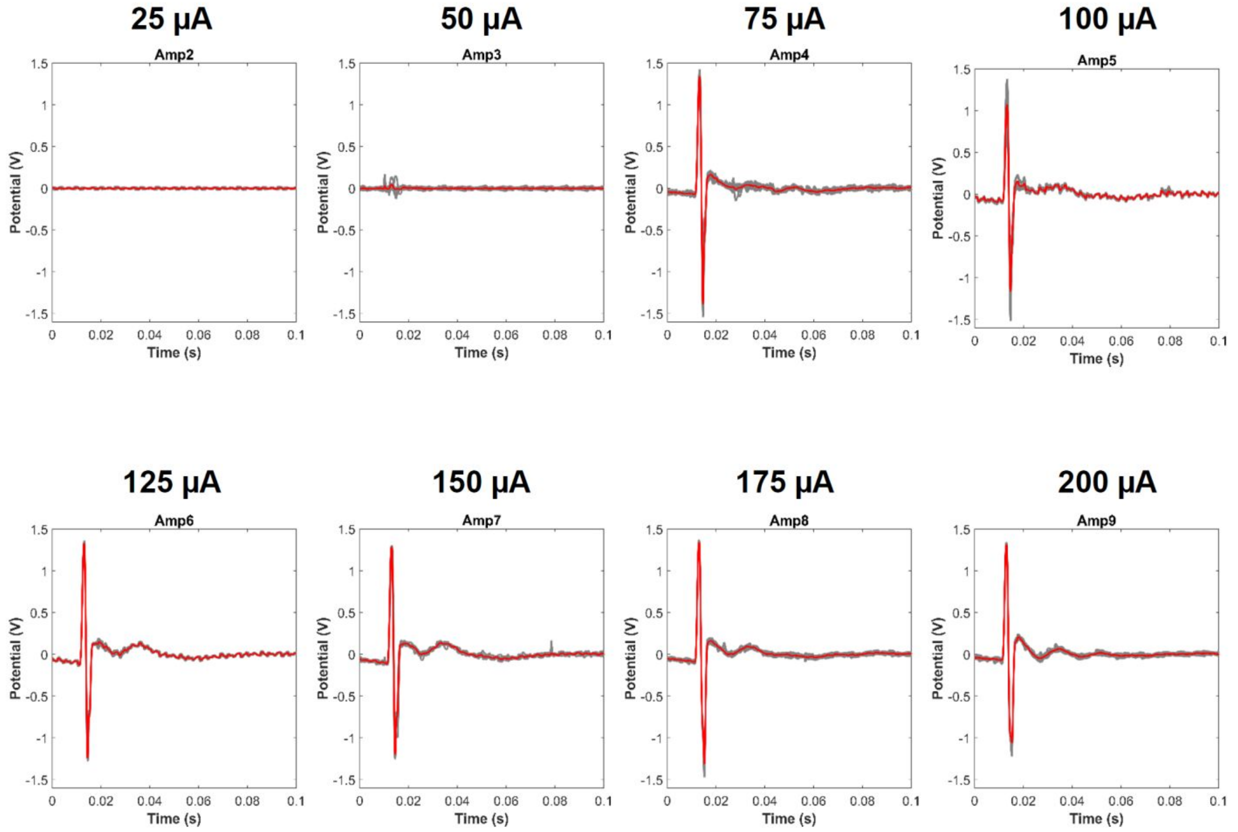

Figure S31. Evoked electromyography response under increasing stimulation amplitudes. The potential measured as a function of time for bipolar stimulation with a pair of 300 µm electrodes and EMG recorded with a 300 µm electrode. Grey plots denote the individual pulses (n=10), and the red plots denote the average of individual traces.

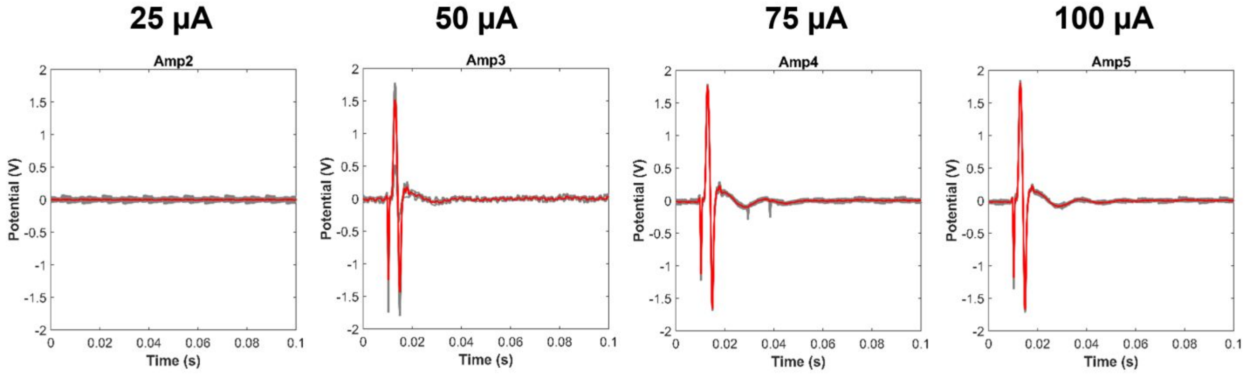

Figure S32. The potential measured as a function of time for bipolar stimulation with a pair of 100 µm electrodes and EMG recorded with a 100 µm electrode. Grey plots denote the individual pulses (n=10), and the red plots denote the average of individual traces.

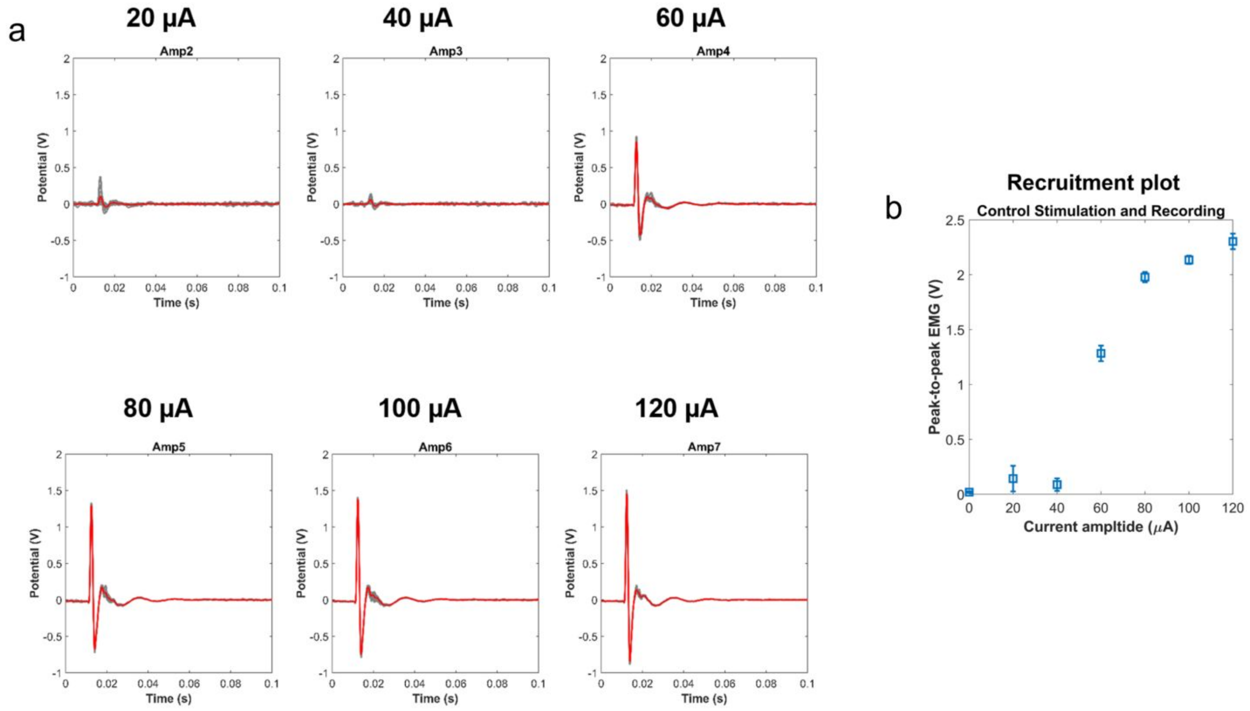

Figure S33. a) Potential measured as a function of time for bipolar stimulation with a commercially available W electrode and EMG recorded with a commercially available electrode. Grey plots denote the individual pulses (n=10) and the red plots denote the average of individual traces. b) Measured peak-to-peak evoked EMG response as a function of increasing stimulation currents.

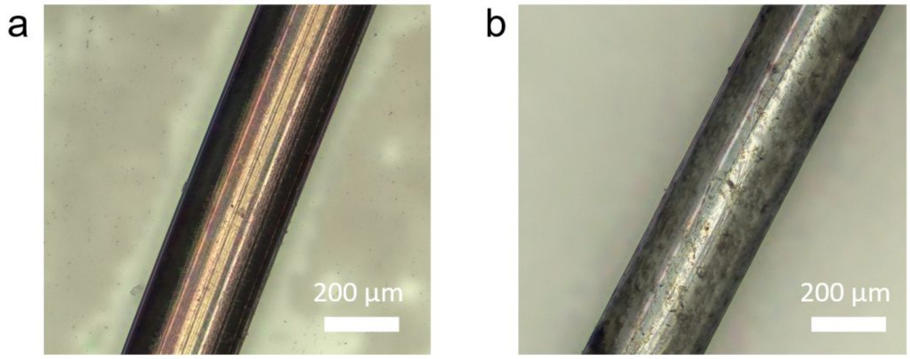

Figure S34. Optical images of 300 μm-diameter nylon filaments coated with a) 70 mg/mL V_2C solution (average flake size 500 nm, zeta potential – 36.4 eV) and b) a solution of 30 mg/mL graphene oxide (GO) and Ti_3C_2 in a 1:1 weight percentage ratio.

Table S1 Linear resistance and filament conductivity of the dip coating parametric study.

Diameter (µm)	Concentration (mg/ml)	Speed (mm/s)	Resistance (Ω/cm)	SD (Ω/cm)	Fiber conductivity, including substrate (S/cm)	SD (S/cm)
100	20	1	2405.0	530.6	5.30	1.17
100	20	5	5444.0	1506.7	2.34	0.65
100	20	15	2648.0	240.6	4.81	0.44
100	40	1	3003.0	637.4	4.24	0.90
100	40	5	1382.0	211.3	9.22	1.41
100	40	15	1242.0	116.3	10.26	0.96
100	80	1	518.4	607.4	24.57	28.79
100	80	5	270.5	58.1	47.09	10.12
100	80	15	159.9	24.4	79.67	12.15
200	20	1	1262.5	132.8	2.52	0.27
200	20	5	513.5	80.4	6.20	0.97
200	20	15	271.6	11.2	11.73	0.48
200	40	1	1008.7	145.9	3.16	0.46
200	40	5	403.6	60.1	7.89	1.18
200	40	15	193.9	40.2	16.42	3.40
200	80	1	44.7	12.0	71.33	19.14
200	80	5	104.6	9.9	30.45	2.87
200	80	15	60.0	5.1	53.09	4.49
300	20	1	1408.0	266.5	1.01	0.19
300	20	5	505.4	97.0	2.80	0.54
300	20	15	169.0	20.1	8.37	0.99
300	40	1	907.8	122.2	1.56	0.21
300	40	5	282.6	44.5	5.01	0.79
300	40	15	100.5	6.9	14.09	0.97
300	80	1	36.3	7.8	39.01	8.43
300	80	5	49.4	4.5	28.65	2.58
300	80	15	46.2	4.4	30.64	2.93

Table S2. Electrical conductivity of MXene coatings on 300 μm-diameter filaments.

Specification of coated nylon filament	Resistance (Ω/cm)	Resistance SD (Ω/cm)	Geometrical factor	MXene coating thickness (nm)	Conductivity of the MXene coating (S/cm)	Conductivity SD (S/cm)
300 μm - 110 mg/ml - 15 mm/s	9.3	1.1	10.6	1600	7093	819
300 μm - 10 mg/ml- 15 mm/s	681.0	43.6	10.6	40	3891	249

Table S3. Resistance, conductivity and density comparison between MXene-coated nylon filaments and commercial silver-plated nylon filaments.

Conductive filaments	Resis-tance (Ω/cm)	SD (Ω/cm)	Filament conduc-tivity (S/cm)	SD (S/cm)	Coating thickness (nm)	Linear coating loading (mg/cm)	Linear pristine nylon density (mg/cm)	Active material (wt%)	Ref.
100 μm nylon coated with 110 mg/ml $Ti_3C_2T_x$ at 15 mm/s	41.9	6.7	304.0	48.6	440	0.005	0.087	5.3%	This work
300 μm nylon coated with 110 mg/ml $Ti_3C_2T_x$ at 15 mm/s	9.3	1.1	152.2	18.0	1800	0.059	0.781	7.1%	This work
80 μm nylon plated with silver (Shieldex®)	~200	-	99.5	-	-	0.009	0.057	13.6%	[7]
100 μm nylon plated with silver (Shieldex®)	~100	-	127.4	-	-	0.010	0.090	10.0%	[8]
125 μm nylon plated with silver (Shieldex®)	~100	-	81.5	-	-	0.025	0.165	13.2%	[9]

Table S4 Bending stiffness of MXene-nylon-Parylene C fiber electrodes compared to representative fiber electrodes in the literature that are made of other materials.

Electrode	Diameter or thickness (µm)	Bending stiffness ($N.m^2$)	Stiffness including insulation (Y/N)	Ref.
Pristine Nylon filament	300	9.0×10^{-7}	N	This work
Pristine Nylon filament	100	1.1×10^{-8}	N	This work
Pristine Nylon filament	28	6.8×10^{-11}	N	This work
Nylon/MXene N300-M110-S15	303.2	1.3×10^{-6}	N	This work
Nylon/MXene N100-M110-S15	100.9	1.5×10^{-8}	N	This work
Nylon/MXene N28-M110-S15	28.9*	1.5×10^{-10}	N	This work
Nylon/MXene/Parylene C N300-M110-S15-PC	323.2	1.6×10^{-6}	Y	This work
Nylon/MXene/Parylene C N100-M110-S15-PC	120.9	3.2×10^{-8}	Y	This work
Nylon/MXene/Parylene C N28-M110-S15-PC	48.9*	9.3×10^{-10}	Y	This work
Si probe	25	2.5×10^{-8}	Y	[10]
Platinum wire	25	4.0×10^{-9}	N	[11]
Tungsten wire	25	1.0×10^{-8}	N	[11]
Tungsten wire	50	1.2×10^{-7}	N	[12]
Bare carbon fiber	5	2.7×10^{-11}	N	[12]
CNT fiber	22	1.1×10^{-9}	N	[13]
CNT fiber	12	3.3×10^{-10}	N	[13]

* For 28 µm electrodes, we assumed the same coating thickness as 100 µm diameter electrodes, even though a thinner coating is anticipated.

Table S5 Impedance, CICc and CSCc of MXene-nylon-Parylene C fiber electrodes compared to the best of fiber electrodes in literature.

Electrode	Area used for normalization (μm^2)	CSCc (mC/cm^2)	CICc (mC/cm^2)	1 kHz |Z| in 1X PBS (kΩ)	Water win-dow	Ref.

Nylon/MXene /Parylene C 300 µm-110 mg/ml -15 cm/s	Total exposed 72202 ± 27 Active only 1516 ± 0.6	46.2 ± 7.7 at 100 mV/s by total 2202 ± 366 by active only	0.44 ± 0.07 by total 21.2 ± 3.3 by active only	4.70 ± 0.55	-1.3 to 0.4	This work
Nylon/MXene /Parylene C 100 µm-110 mg/ml -15 cm/s	Total exposed 7993 ± 4.7 Active only 138 ± 0.08	59.2 ± 4.7 at 100 mV/s by total 3409.3 ± 268.9 by active only	0.68 ± 0.19 by total 39.0 ± 11.1 by active only	14.0 ± 3.1	-1.3 to 0.4	This work
Nylon/MXene /Parylene C 300 µm-10 mg/ml -15 cm/s	Total exposed 70720 ± 1 Active only 34.1 ± 0	-	-	-	-	This work
Bare carbon fiber (CF)	Active only	-	-	1000	-0.6 to 0.4	14
Iridium oxide film (EIROF) coated CF	Total ≈ active	17	25	57	-0.6 to 0.6	15
Graphene fiber /parylene C	Total ≈ active 169 ± 25	798 ± 110 at 10 mV/s	8.9 ± 1.3	51.5 ± 0.2	-1.0 to 0.9	16
Graphene fiber/ Pt /parylene C	Total ≈ active 169 ± 25	946 ± 140 at 10 mV/s	10.5 ± 1.5	11.2 ± 0.2	-1.0 to 0.9	16
Graphene fiber /parylene C	Total ≈ active 749 ± 93	200 ± 25 at 10 mV/s	4.7 ± 0.6	37.9 ± 0.2	-1.0 to 0.9	16
Graphene fiber/ Pt /parylene C	Total ≈ active 749 ± 93	361 ± 45 at 10 mV/s	8.0 ± 1.0	5.2 ± 0.2	-1.0 to 0.9	16
CNT fiber	Total ≈ active 1450	372 ± 56 at 100 mV/s	6.52	14.1 ± 0.4	-0.6 to 0.8	17
Si fiber	Total ≈ active	-	-	980	-	18
PEDOT:PSS deposited Si fiber	Total ≈ active	-	-	130	-	18
Amorphous silicon carbide (a-SiC) fiber	Total ≈ active	-	-	128	-	19
Pt fiber	Total ≈ active 7850	1.2 at 50 mV/s	0.2	54.1	-0.6 to 0.8	20
PtIr fiber	Total ≈ active 17000	1.2 ± 0,08 at 100 mV/s	0.15	26.6	-0.6 to 0.8	17
PEDOT:PSS deposited on PtIr fiber	Total ≈ active 4500	123 at 1V/s	2.92	18	-0.6 to 0.8	21
PPy/CNT deposited Pt fiber	Total ≈ active 12434	1244 at 50 mV/s	7.5	2.06	-0.6 to 0.6	20
Multi-material thermal drawn fiber	Total	-	-	223.9 ± 36.6	-	22

References

(1) Shuck, C. E.; Sarycheva, A.; Anayee, M.; Levitt, A.; Zhu, Y.; Uzun, S.; Balitskiy, V.; Zahorodna, V.; Gogotsi, O.; Gogotsi, Y. Scalable Synthesis of $Ti_3C_2T_x$ MXene. *Adv. Eng. Mater.* **2020**, *22* (3), 1901241. https://doi.org/10.1002/adem.201901241.
(2) Lu, L.; Fu, X.; Liew, Y.; Zhang, Y.; Zhao, S.; Xu, Z.; Zhao, J.; Li, D.; Li, Q.; Stanley, G. B.; Duan, X. Soft and MRI Compatible Neural Electrodes from Carbon Nanotube Fibers. *Nano Lett.* **2019**, *19* (3), 1577–1586. https://doi.org/10.1021/acs.nanolett.8b04456.
(3) *Overview of materials for Nylon 66/6*. https://www.matweb.com/search/datasheet_print.aspx?matguid=26386631ec1b49eeba62c80a49730dc4 (accessed 2023-07-29).
(4) Zhang, J.; Kong, N.; Uzun, S.; Levitt, A.; Seyedin, S.; Lynch, P. A.; Qin, S.; Han, M.; Yang, W.; Liu, J.; Wang, X.; Gogotsi, Y.; Razal, J. M. Scalable Manufacturing of Free-Standing, Strong Ti3C2Tx MXene Films with Outstanding Conductivity. *Adv. Mater.* **2020**, *32* (3), 2001093.
(5) MuGuinnesss, M. *Parylene C Datasheet*. https://www.hzo.com/blog/parylene-c-datasheet-pdf-download/ (accessed 2023-07-29).
(6) Zhang, J.; Uzun, S.; Seyedin, S.; Lynch, P. A.; Akuzum, B.; Wang, Z.; Qin, S.; Alhabeb, M.; Shuck, C. E.; Lei, W.; Kumbur, E. C.; Yang, W.; Wang, X.; Dion, G.; Razal, J. M.; Gogotsi, Y. Additive-Free MXene Liquid Crystals and Fibers. *ACS Cent. Sci.* **2020**, *6* (2), 254–265. https://doi.org/10.1021/acscentsci.9b01217.
(7) *Shieldex® 80 µ Monofil*. Shieldex® – Metallized Technical Textiles. https://www.shieldex.de/en/products/shieldex-80-%ce%bc-monofil/ (accessed 2023-09-16).
(8) *Shieldex® 100 µ Monofil*. Shieldex® – Metallized Technical Textiles. https://www.shieldex.de/en/products/shieldex-100-%ce%bc-monofil/ (accessed 2023-09-16).
(9) *Shieldex® 125 µ Monofil*. Shieldex® – Metallized Technical Textiles. https://www.shieldex.de/en/products/shieldex-125-%ce%bc-monofil/ (accessed 2023-09-16).
(10) Subbaroyan, J.; Martin, D. C.; Kipke, D. R. A Finite-Element Model of the Mechanical Effects of Implantable Microelectrodes in the Cerebral Cortex. *J. Neural Eng.* **2005**, *2* (4), 103–113. https://doi.org/10.1088/1741-2560/2/4/006.
(11) Vitale, F.; Vercosa, D. G.; Rodriguez, A. V.; Pamulapati, S. S.; Seibt, F.; Lewis, E.; Yan, J. S.; Badhiwala, K.; Adnan, M.; Royer-Carfagni, G.; Beierlein, M.; Kemere, C.; Pasquali, M.; Robinson, J. T. Fluidic Microactuation of Flexible Electrodes for Neural Recording. *Nano Lett.* **2018**, *18* (1), 326–335. https://doi.org/10.1021/acs.nanolett.7b04184.
(12) Hejazi, M.; Tong, W.; Ibbotson, M. R.; Prawer, S.; Garrett, D. J. Advances in Carbon-Based Microfiber Electrodes for Neural Interfacing. *Front. Neurosci.* **2021**, *15*, 658703. https://doi.org/10.3389/fnins.2021.658703.
(13) Adnan, M.; Pinnick, R. A.; Tang, Z.; Taylor, L. W.; Pamulapati, S. S.; Carfagni, G. R.; Pasquali, M. Bending Behavior of CNT Fibers and Their Scaling Laws. *Soft Matter* **2018**, *14* (41), 8284–8292. https://doi.org/10.1039/C8SM01129J.
(14) Guitchounts, G.; Markowitz, J. E.; Liberti, W. A.; Gardner, T. J. A Carbon-Fiber Electrode Array for Long-Term Neural Recording. *J. Neural Eng.* **2013**, *10* (4), 046016. https://doi.org/10.1088/1741-2560/10/4/046016.

(15) Deku, F.; Joshi-Imre, A.; Mertiri, A.; Gardner, T. J.; Cogan, S. F. Electrodeposited Iridium Oxide on Carbon Fiber Ultramicroelectrodes for Neural Recording and Stimulation. *J. Electrochem. Soc.* **2018**, *165* (9), D375–D380. https://doi.org/10.1149/2.0401809jes.
(16) Wang, K.; Frewin, C. L.; Esrafilzadeh, D.; Yu, C.; Wang, C.; Pancrazio, J. J.; Romero-Ortega, M.; Jalili, R.; Wallace, G. High-Performance Graphene-Fiber-Based Neural Recording Microelectrodes. *Adv. Mater.* **2019**, *31* (15), 1805867. https://doi.org/10.1002/adma.201805867.
(17) Vitale, F.; Summerson, S. R.; Aazhang, B.; Kemere, C.; Pasquali, M. Neural Stimulation and Recording with Bidirectional, Soft Carbon Nanotube Fiber Microelectrodes. *ACS Nano* **2015**, *9* (4), 4465–4474. https://doi.org/10.1021/acsnano.5b01060.
(18) Ludwig, K. A.; Uram, J. D.; Yang, J.; Martin, D. C.; Kipke, D. R. Chronic Neural Recordings Using Silicon Microelectrode Arrays Electrochemically Deposited with a Poly(3,4-Ethylenedioxythiophene) (PEDOT) Film. *J. Neural Eng.* **2006**, *3* (1), 59–70. https://doi.org/10.1088/1741-2560/3/1/007.
(19) Deku, F.; Ghazavi, A.; Cogan, S. F. Neural Interfaces Based on Amorphous Silicon Carbide Ultramicroelectrode Arrays. *Bioelectron. Med.* **2018**, *1* (3), 185–200. https://doi.org/10.2217/bem-2018-0006.
(20) Lu, Y.; Li, T.; Zhao, X.; Li, M.; Cao, Y.; Yang, H.; Duan, Y. Y. Electrodeposited Polypyrrole/Carbon Nanotubes Composite Films Electrodes for Neural Interfaces. *Biomaterials* **2010**, *31* (19), 5169–5181. https://doi.org/10.1016/j.biomaterials.2010.03.022.
(21) Venkatraman, S.; Hendricks, J.; King, Z. A.; Sereno, A. J.; Richardson-Burns, S.; Martin, D.; Carmena, J. M. In Vitro and In Vivo Evaluation of PEDOT Microelectrodes for Neural Stimulation and Recording. *IEEE Trans. Neural Syst. Rehabil. Eng.* **2011**, *19* (3), 307–316. https://doi.org/10.1109/TNSRE.2011.2109399.
(22) Garwood, I. C.; Major, A. J.; Antonini, M.-J.; Correa, J.; Lee, Y.; Sahasrabudhe, A.; Mahnke, M. K.; Miller, E. K.; Brown, E. N.; Anikeeva, P. Multifunctional Fibers Enable Modulation of Cortical and Deep Brain Activity during Cognitive Behavior in Macaques. *Sci. Adv.* **2023**, *10* (09), 511302.
